# Marine Fish-Derived ACE-Inhibitory Peptides: Preparation via Enzymatic Hydrolysis and Their In Vitro Activity

**DOI:** 10.3390/md24100333

**Published:** 2026-09-24

**Authors:** Yufei Zhao, Yongchang Su, Shuilin Cai, Bei Chen, Kun Qiao, Zhiyu Liu

**Affiliations:** 1 School of Basic Medicine and Clinical Pharmacy, China Pharmaceutical University, No. 639, Longmian Avenue, Jiangning District, Nanjing 211198, China; fiona040510@gmail.com; 2 Key Laboratory of Cultivation and High-Value Utilization of Marine Organisms in Fujian Province, Fujian Collaborative Innovation Center for Exploitation and Utilization of Marine Biological Resource, Fisheries Research Institute of Fujian, Xiamen 361013, China

**Keywords:** marine fish, ACE-inhibitory peptides, enzymatic hydrolysis, purification, in vitro activity

## Abstract

Hypertension constitutes a major global public health burden. Although angiotensin-converting enzyme (ACE) inhibitors are beneficial, their adverse effects have prompted the search for safer natural alternatives. ACE-inhibitory peptides have drawn extensive research attention, attributed to their low toxicity and high bioavailability. This article reviews current advances regarding the preparation, separation, purification, and in vitro activity evaluation of these peptides obtained from different marine fish species. Raw material sources are classified into major commercial species and minor or cultured species, highlighting the influence of species and tissue origin on peptide bioactivity. Enzymatic hydrolysis, particularly single-enzyme and dual-enzyme approaches, is the main preparation method. Subsequent separation and purification, involving ultrafiltration, gel filtration chromatography, and reversed-phase high-performance liquid chromatography (RP-HPLC), are crucial for enriching bioactive fractions and identifying novel peptide sequences. Also, the classical hippuryl-histidyl-leucine (HHL)-high-performance liquid chromatography (HPLC) assay remains the standard for the evaluation of in vitro activity using IC_50_ values. Future work emphasizes integrated in vitro and in vivo validation, AI-assisted screening, and the sustainable utilization of processing by-products to advance marine peptides as novel antihypertensive agents.

## 1. Introduction

Hypertension acts as a critical risk factor: it causes severe complications such as cardiovascular diseases (CVDs), stroke, and renal failure. As stated in the hypertension report issued by World Health Organization (WHO) in 2023, around 1.28 billion adults aged 30–79 years across the whole world suffer from this chronic disease [1]. The main factors contributing to the high incidence of hypertension are accelerated urbanization, changes in lifestyle, and population aging [2]. Currently, the clinical management of hypertension relies mainly on angiotensin-converting enzyme (ACE) inhibitors, calcium channel blockers (CCBs) and Angiotensin II Receptor Blockers (ARBs) [3]. ACE is a zinc-dependent metallopeptidase that acts within the renin–angiotensin system (RAS) and plays a key role in regulating systemic blood pressure. Currently, a variety of artificially synthesized ACE blockers, including lisinopril, captopril, alacepril, and enalapril, have been extensively adopted to treat primary hypertension and heart failure [4,5]. Although these synthetic antihypertensive drugs have definite therapeutic efficacy, some adverse reactions, including headache, hypotension, and allergic reactions, could occur, and may even lead to renal impairment in severe cases [6]. They are also frequently accompanied by other adverse effects, including persistent cough, dysgeusia, and skin rashes [6,7], which severely impair patients’ quality of life. Therefore, the development of safe and efficient natural antihypertensive alternatives with few adverse effects and suitability for long-term administration has become a major focus in hypertension research [8]. Against this research background, bioactive peptides extracted from food sources have emerged as highly promising natural alternative materials for blood pressure reduction, owing to their advantages of high bioavailability, few adverse effects after long-term administration, and additional health-promoting functions [9].

Marine food-derived ACE-inhibitory peptides are sourced from different sources, including mollusks, fish, crustaceans, shellfish, and algae. Due to their unique protein profiles and amino acid compositions, marine organisms are considered valuable sources for developing natural ACE-inhibitory peptides, and have drawn considerable research attention [10,11]. In 1996, Seki et al. [12] reported that marine-derived protein peptides showed higher ACE-inhibitory activity than those derived from livestock, poultry, and milk proteins. To date, researchers have successfully separated and characterized novel ACE-inhibitory peptides from many types of fish species, including tuna, salmon, cod, mackerel, large yellow croaker, anglerfish, and pufferfish [13]. Short-chain peptides with multiple hydrogen bonds and hydrophobic groups are frequently found in fish-derived proteins, exhibiting the advantages in binding to the ACE active site [14]. Therefore, marine-derived ACE-inhibitory peptides can serve as alternative therapeutic agents for blood pressure control [15]. Although they have lower ACE-inhibitory activity relative to captopril, they undoubtedly remain a hot research topic in the field of antihypertensive drug development [13].

This paper summarizes in vitro activity evaluation methods, fish raw material sources, enzymatic hydrolysis preparation methods, separation and purification methods, and the ACE activity of peptides and their derived products. It aims to provide theoretical guidance for developing natural marine fish-derived ACE-inhibitory peptides for novel antihypertensive drugs.

Literature search strategy: The literature covered in this review was collected through systematic searches of the PubMed, Web of Science, Scopus, and Google Scholar databases, complemented by the Chinese-language databases CNKI (China National Knowledge Infrastructure) and Wanfang Data to cover domestic studies. The search terms included “ACE inhibitory peptide”, “angiotensin-I-converting enzyme inhibitory peptide”, “anti-hypertensive peptide”, “marine fish”, “fish protein hydrolysate”, “enzymatic hydrolysis”, and “purification”, as well as combinations of these terms with specific fish names (e.g., tuna, salmon, cod, mackerel, hairtail, yellow croaker, pufferfish, and lizardfish). The search covered publications from 1992 to 2026, with particular emphasis on studies published within the last decade (2016–2026) to reflect the most recent research progress. In addition, the reference lists of key review articles were screened to identify further relevant studies. Studies were considered eligible if they reported the preparation, isolation, purification, sequence identification, or in vitro ACE-inhibitory activity evaluation of peptides derived from marine fish species or their processing by-products. Conference abstracts, non-peer-reviewed articles, and studies on non-fish marine organisms (e.g., mollusks, crustaceans, and algae) were excluded.

## 2. In Vitro Activity Evaluation Methods for ACE-Inhibitory Peptides

In vitro experiments remain the principal and most widely employed method for ACE-inhibitory activity evaluation, and the HHL-HPLC assay is a classical method, which employs hippuryl-his-leu (HHL) as the substrate. Hippuric acid (HA) is produced upon the catalytic action of ACE, and reversed-phase high-performance liquid chromatography (RP-HPLC) with a C18 chromatographic column serves to separate and quantify HA. The ACE inhibition rate is calculated from the HA peak area at 228 nm, and the result is typically expressed as a half-maximal inhibitory concentration (IC_50_) [16]. Due to the advantages of high sensitivity and accurate quantification, this method is commonly recognized as a “gold standard” for measuring ACE activity, and it has been widely adopted to assess the bioactivity of food-derived ACE-inhibitory peptides. The HPLC method has revealed that Captopril, a typically representative synthetic antihypertensive drug, exhibits potent ACE-inhibitory activity with an IC_50_ value expressed in the nanomolar range by HHL-HPLC assay [17,18]. According to the literature [19], ACE-inhibitory peptides with an IC_50_ between 0.27 and 1000 μM have the potential to lower blood pressure. Based on the presenting research findings, ACE-inhibitory activities of food-derived bioactive peptides are mostly quantified in the micromolar (μM) range, and their activities are far lower than that of captopril. Based on the reported IC_50_ values, ACE-inhibitory activity may be classified as extremely low activity (>1000 μM), low activity (500–1000 μM), moderate activity (100–500 μM), high activity (10–100 μM), and extremely high activity (<10 μM).

Because the level of structural characterization determines how the reported activity data should be interpreted, the data extracted from the included studies are classified into three levels of material characterization, distinguished by molecular weight (MW) distribution and the degree of structural resolution. Crude hydrolysates, which are the unfractionated products of enzymatic digestion, exhibit broad MW distributions without peptide sequence identification. For example, bigeye tuna skin hydrolysate contained 60% of peptides below 3000 Da [20], Alaska pollock skin hydrolysate contained 93.08% below 1000 Da [21], and longsnout catfish hydrolysate contained 98.2% below 500 Da [22]. These heterogeneous mixtures are characterized by bulk ACE inhibition rate (%) or composite IC_50_ values. Separated fractions, obtained via ultrafiltration (UF) with defined MWCO membranes (typically 1, 3, 5, and 10 kDa) or gel filtration chromatography (GFC), exhibit progressively narrowed MW ranges. For instance, Atlantic cod skin collagen hydrolysate was refined from <1000 Da (82%) through UF to <500 Da (85%) and then to <500 Da (97%) after GFC [23]; sequential UF with 0.65, 1, 3.5, and 5 kDa membranes narrowed Alaska pollock skin fractions to 400–1708 Da [24]; and UF with a 0.65 kDa membrane yielded a salmon bone fraction with IC_50_ = 0.093 μg/mL [25]. However, these fractions remain peptide mixtures whose composite IC_50_ values may reflect synergistic or antagonistic interactions [26]. Purified peptides, obtained after final RP-HPLC isolation and MS/MS identification, possess discrete MW values, unambiguous amino acid sequences, and individual IC_50_ values—e.g., FPPDVA (644.32 Da, IC_50_ = 87.1 μM) [27], GPAGPSGP (638.67 Da, IC_50_ = 179 μM) [23], and GSAGPAGPSGPRGP (1163.56 Da, IC_50_ = 166 μM) [24]. Only purified peptides provide the structural basis for mechanistic studies, molecular docking, and QSAR modeling. This three-tier classification is applied consistently throughout the manuscript.

## 3. Fish Raw Material Sources of Marine Fish-Derived ACE-Inhibitory Peptides

### 3.1. Major Commercial Fish Species

#### 3.1.1. Scombridae Fish

Scombridae fish, an important group of marine commercial species, are suitable raw materials for ACE-inhibitory peptide production due to their high protein content and balanced amino acid profiles. Among them, tuna and skipjack tuna are two main species, and these have been extensively studied (Figure 1). The by-products generated during tuna processing are valuable raw materials; for example, tuna produces large amounts of by-products, accounting for up to 50% of raw fish weight [28]. Tuna dark muscle contains abundant protein, rich in hydrophobic and basic amino acid residues, a feature that aligns with the typical structural traits of ACE-inhibitory peptides, making it an excellent starting substrate for developing blood pressure-lowering peptide compounds.

Recently, peptides including FPPDVA (645 Da) and KFPWGDGN (919 Da) from albacore tuna (*Thunnus alalunga*) dark muscle showed high ACE-inhibitory activity, with respective IC_50_ values of 87.1 μM and 297.5 μM [27]. A 13-amino acid peptide (WPEAAELMMEVDP, 1581 Da) from bigeye tuna (*Thunnus obesus*) dark muscle exhibited high ACE-inhibitory activity (IC_50_ = 21.6 μM) [29]. Researchers adopted yellowfin tuna (*Thunnus albacares*) muscle as raw material to screen two bioactive peptides STHPHF (724.33 Da) and LTFSY (629.31 Da) (Purified peptide) after enzymatic hydrolysis, whose IC_50_ values were 42.40 ± 0.25 μM and 32.92 ± 0.24 μM, respectively [30]. Similarly, two highly active peptides LTGCP (489.59 Da) and YPKP (503.60 Da) (Purified peptide) were purified from the enzymatic hydrolysate of bluefin tuna (*Thunnus thynnus*) meat, with IC_50_ values reaching 64.3 μM and 139.6 μM, respectively [31]. In contrast to these short peptides, GDLGKTTTVSNWSPPKWKDTP (2482 Da) (Purified peptide), a high-molecular-weight 21-amino acid peptide, was recovered from the proteolytic hydrolysate of bigeye tuna steaks, with an IC_50_ value of only 11.28 μM, representing a relatively long marine fish-derived ACE-inhibitory peptide with extremely high activity [32]. These findings indicate that the molecular weights of tuna-derived ACE peptides vary widely in sequence length, mainly ranging from tetrapeptides to heptapeptides with high ACE-inhibitory activity.

Skipjack tuna, another member of the family Scombridae, has benefits of high production yield, abundant by-products, and low cost, giving it potential industrial development value. WGESF, IKSW, YSHM, and WSPGF have been isolated from the hydrolysate of skipjack tuna (*Katsuwonus pelamis*) roe, and their molecular weights were in the range of 532.58 and 624.63 Da, and their IC_50_ values were higher than 900 μM, showing relatively low ACE activity [28]. Similarly, 14 short peptides (146.15 Da–977.24 Da) were obtained from skipjack tuna dark muscle hydrolysate, exhibiting a wide range of IC_50_ values (546.04–18,556.28 μM). Among them, three peptides of MKKS, MWN and LPRS exhibited relatively high activity with IC_50_ values of 546.04 μM, 729.65 μM and 1049.71 μM, respectively [33]. Based on the above, most skipjack tuna peptides produced by conventional protease hydrolysis are from dipeptides to pentapeptides. Although their molecular weights are small, their ACE-inhibitory activity is generally low. However, Yokoyama et al. [34] successfully obtained eight extremely highly active peptides from the thermolysin hydrolysate of dried skipjack tuna. Among them, four peptides of IWHHT, IVGPRPRHQG, ALPHA, and FQP were derived from actin with IC_50_ values of 5.1, 6.2, 10, and 12 μM, respectively, showing extremely high inhibitory activity. IKPLNY derived from myosin possessed an IC_50_ of 43 μM, while DYGLYP obtained from fibronectin reached an IC_50_ of 62 μM; both exhibited high inhibitory activity similar to the activity of DMIPAQK (45 μM) purified from the hot water extract of dried skipjack tuna.

#### 3.1.2. Salmonids

Salmonids are also typical marine fish species for producing ACE-inhibitory peptides. An 8-amino acid peptide FCLYELAR (1041.2 Da) was purified from the enzymatic hydrolysate of Atlantic salmon (*Salmo salar*) bone, with an IC_50_ value of 0.25 ± 0.02 μM, demonstrating extremely high activity [25]. Similarly, three peptides of FNVPLYE, VWDPPKFD, and FEDYVPLSCF were identified from the proteolytic hydrolysate of Atlantic salmon pectoral fin (Crude hydrolysate) with IC_50_ values of 7.72, 9.10, and 10.77 μM, respectively [35]. In contrast, three short peptides (GR, RER, GPR) from the collagen hydrolysate of Atlantic salmon skin were identified, and they all showed IC_50_ values higher than 2500 μM [36]. By comparison, chum salmon (*Oncorhynchus keta*) skin hydrolysate yielded peptide GLPLNLP with a lower IC_50_ value of 18.7 μM, and its activity was far superior to collagen peptides from Atlantic salmon skin [37].

Overall, the peptides recovered from proteolyzed salmon meat, fin, and bone have higher activity than those from skin collagen peptides, and the activity of collagen peptides from Atlantic salmon skin is generally extremely low. The activity of peptides derived from different parts of the same salmon individual varies greatly. In summary, peptides derived from salmonids generally exhibit high ACE-inhibitory activity.

#### 3.1.3. Gadidae Fish

With the advantages of large production yield, abundant by-products, and low cost, Gadidae fish species seem to be an important raw material for collagen peptide preparation in the aquatic processing industry. Atlantic cod (*Gadus morhua*) and Alaska pollock (*Gadus chalcogrammus*) are the two main representative commercial species, and their derived peptides differ considerably in ACE activity. MLGP (516.54 Da), MWW (521.63 Da), and GPAGPSGP (638.67 Da) were obtained from the hydrolysate of Atlantic cod (*Gadus morhua*) skin with respective IC_50_ values of 683.1, 497.6, and 179 μM, showing moderate ACE-inhibitory activity [23]. In recent years, many peptides were isolated from skin hydrolysates of Alaska pollock (*Gadus chalcogrammus*), including GPAGPSGP (IC_50_ = 179.5 ± 12 μM) [38], GSAGPAGPSGPRGP (166 μM), LGDARNSPAPP (177 μM) [24] and GPLGVP (105.8 μM) [39], and these peptides exhibited higher inhibitory activity than that from Alaska pollock (*Gadus chalcogrammus*). With the advantages of sufficient raw material supply and cost-effectiveness, Alaska pollock has become the mainstream raw material for developing cod-derived ACE-inhibitory peptides. However, strategies are still needed to further improve their ACE-inhibitory activity.

### 3.2. Minor Commercial Fish Species

#### 3.2.1. Small- and Medium-Sized Marine Fish Species

Apart from major commercial fish species, studies have also investigated small- and medium-sized marine fish such as sardines and snakehead murrel, and they possessed short peptides with high ACE-inhibitory activity. Six unreported novel tripeptides and tetrapeptides of FIGR, FILR, FQRL, FRAL, KLF, and KFL were screened from the enzymatic hydrolysate of European pilchard (*Sardina pilchardus*), with molecular weights of 480.56, 463.53, 459.52, 492.58, 347.42, and 347.42 Da and IC_50_ values of 1.25, 2.18, 3.35, 1.34, 1.76, and 2.56 μM, respectively, all showing extremely high inhibitory activity [40]. Similarly, a heptapeptide VPAAPPK and an octapeptide NGTWFEPP were isolated from the myofibrillar protein hydrolysate of striped snakehead (*Channa striatus*) with IC_50_ values of 0.45 μM and 0.63 μM, respectively, ranking among the most potent marine fish-derived ACE-inhibitory peptides [41]. Additionally, dipeptide Ala-Pro produced by the degradation of Japanese pilchard (*Sardinops melanostictus*) was reported to have a spatial structure similar to the antihypertensive drug captopril, representing a typical natural and highly efficient ACE-inhibitory short peptide, with higher activity than many reported aquatic-derived short peptides [42].

#### 3.2.2. Offshore Cultured Commercial Fish Species

Common offshore cultured commercial fish species also exhibit a good potential for preparing ACE-inhibitory peptides, and they mainly covered the Sciaenidae family and *Tetraodontiformes* order. Six peptides of LYDHLGK, IPYADFK, INEMLDTK, IHFGTTGK, NWPWMK, and FYEPFM were obtained by hydrolyzing the myosin of large yellow croaker (*Larimichthys crocea*) with IC_50_ values of 9.41 ± 0.24 μM, 0.63 ± 0.09 μM, 6.53 ± 0.21 μM, 5.34 ± 0.15 μM, 6.23 ± 0.34 μM, and 10.26 ± 0.21 μM, respectively, all exhibiting extremely high inhibitory activity [43]. Similarly, two tripeptides of WAR and WQR with molecular weights of 431.52 Da and 488.58 Da were separated from the titin hydrolysate of large yellow croaker (*Larimichthys crocea*), and they demonstrated high inhibitory activity with low IC_50_ values of 31.2 ± 0.8 μM and 231.33 ± 0.02 μM, respectively [44]. Furthermore, HDHSE and GENSQ were isolated from small yellow croaker (*Larimichthys polyactis*) muscle with IC_50_ values of 38.74 μM and 65.377 μM, respectively [45]. In addition, a novel highly active peptide PPLLFAAL (841.05 Da) was obtained from the enzymatic hydrolysate of tawny puffer (*Takifugu flavidus*) skin with an IC_50_ value of 28 μM [46]. Existing experimental data prove ACE-inhibitory peptides from offshore cultured commercial fish species have great development and application potential.

#### 3.2.3. Other Minor Marine Fish Species

In addition to the aforementioned species, other minor marine fish species such as cobia, pacific saury, hairtail, chub mackerel, anglerfish, bighead croaker, and lizardfish also have been widely studied, and activities of peptides obtained from different fish species and tissues varies greatly. Four peptides were identified from cobia (*Rachycentron canadum*) skin hydrolysate, among them, IWW (388.45 Da) and AWW (374.42 Da) showed extremely high inhibitory activity of IC_50_ value of 0.51 and 9.40 μM, respectively, WL (261.31 Da) with an IC_50_ value of 26.80 μM showed a high inhibitory activity, and WAA (331.35 Da) showed moderate activity [47]. Similarly, a synthesized peptide (LEPWR, 758.85 Da) from hydrolysate of Pacific saury (*Cololabis saira*) showed high activity with IC_50_ value of 99.5 μM [48]. Additionally, two highly active ACE-inhibitory peptides of ANSEVAQWR and EALVSQLTR were purified from the myosin hydrolysate of hairtail (*Trichiurus lepturus*) with IC_50_ values of 89.58 μM and 91.48 μM [49]. Two peptides of PLITT (543.63 Da) and LTPFT (564.66 Da) were purified from chub mackerel (*Scomber japonicus*) hydrolysate, and PLITT exhibited high activity (IC_50_ = 48.73 ± 7.59 μM) [50]. More recently, ten novel ACE-inhibitory peptides were identified from chub mackerel muscle papain hydrolysate through virtual screening combined with network pharmacology analysis, among which APFLAG (IC_50_ = 69.45 ± 2.93 μM) exhibited mixed-type inhibition [51]. Five potential ACE-inhibitory peptides of VELGV, SVAPL, SLAGLP, TDIGVAGI, and LGGGPI were obtained through virtual screening from the proteolytic hydrolysate of hairtail meat (*Trichiurus lepturus*) [52].

Collagen peptides from bighead croaker and anglerfish swim bladders generally have extremely low inhibitory effects. SEGPK, FDGPY, and SPGPW were obtained from the swim bladder of anglerfish (*Lophius litulon*), all molecular weights were below 600 Da, but their IC_50_ values were higher than 1200 μM, showing extremely low ACE-inhibitory activity [8]. Similarly, seven peptides from bighead croaker (*Miichthys miiuy*) swim bladders had extremely low activity by showing IC_50_ values higher than 1400 μM, except for SPYGF (IC_50_ = 649.58 μM) [53]. The activity of peptides obtained from different tissues of the same fish species also varies. Peptides from lizardfish meat exhibited higher inhibitory activity than that from scale gelatin peptides. For example, peptide AGPPGSDGQPGAK (1137.3 Da) obtained from the scale gelatin of lizardfish (*Saurida elongata*) achieved moderate inhibitory activity (IC_50_ = 420 ± 20 μM) [54]. Furthermore, SPRCR, GMLCAP and VYP were screened from lizardfish meat hydrolysate and showed high inhibitory activity, with an IC_50_ value of 41 ± 1 μM [55], 47.3 ± 1.4 μM [56] and 10 μM [57], respectively. More recently, a pentapeptide (RVCLP) identified from lizardfish (*Saurida elongata*) muscle was characterized as a non-competitive ACE inhibitor and could be absorbed across the intestinal epithelium via the paracellular route [58].

In a conclusion, ACE-inhibitory activities of peptides vary from fish to fish. Even for the same fish species, distinctly different activities of ACE-inhibitory peptides can be observed among different tissues and protein fractions. This discrepancy associates closely with the differences in protein profiles and amino acid compositions of different fish raw materials. Therefore, the selection of fish raw materials is a critical step for preparing high-inhibitory-activity ACE-inhibitory peptides.

## 4. Enzymatic Hydrolysis Preparation Methods of Marine Fish-Derived ACE-Inhibitory Peptides

Marine fish-derived ACE-inhibitory peptides can be produced through several approaches, including enzymatic hydrolysis, chemical hydrolysis, microbial fermentation, and simulated gastrointestinal digestion [13]. Currently, the enzymatic hydrolysis method is the most widely adopted and powerful approach to produce bioactive peptides. This method demonstrates the benefits of mild reaction conditions, safety, the structural stability of peptides, high process controllability, and reproducibility [13], and it has become the mainstream method for the preparation of marine-derived ACE-inhibitory peptides at this moment [11]. Each protease possesses unique substrate cleavage specificity; therefore, the resulted hydrolysates show differences in ACE-inhibitory activity, hydrolysis degree, and peptide yield [20].

Up to now, single-enzyme and dual-enzyme combination hydrolysis approaches have been widely adopted for protein hydrolysate preparation (Figure 2). ACE-inhibitory activity serves as the main indicator for protease screening and the degree of hydrolysis as a supplementary index, followed by the parameter optimization of the enzymatic hydrolysis process. Dual-enzyme hydrolysis can be carried out by either simultaneous or stepwise hydrolysis. Microbial proteases, animal proteases, and their combinations are commonly adopted for preparing ACE-inhibitory peptides. The differential cleavage sites of diverse proteases enable fish proteins to undergo sufficient hydrolysis, and potent ACE-inhibitory peptides with varying molecular weights and amino acid sequences can be obtained by subsequent separation and purification techniques. The enzymatic hydrolysis, separation, purification, and identification methods for each reported peptide, together with the key parameters, are systematically compiled in Table 1.

### 4.1. Single-Enzyme Hydrolysis

Single-enzyme hydrolysis refers to fish protein hydrolysis using one type of protease. For example, by-products of Spanish mackerel were directly hydrolyzed with a single alkaline protease, yielding a hydrolysate with an ACE inhibition rate of 45.60% [62]. Similarly, chub mackerel (*Scomber japonicus*) protein was directly hydrolyzed with a single alkaline protease to obtain ACE-inhibitory peptides [50]. In contrast, trypsin was used to hydrolyze Atlantic salmon (*Salmo salar*) bone protein, and, after process optimization, the hydrolysate showed an IC_50_ value of 7.14 ± 0.05 μg/mL at a hydrolysis degree of 7.36 ± 0.69% [25]. For lizardfish (*Saurida elongata*) protein, neutral protease was adopted for hydrolysis; after process optimization and ultrafiltration treatment, the ACE inhibition rate of the hydrolysate reached 84.0% [55]. On this basis, ACE-inhibitory peptides from lizardfish protein using neutral protease were also prepared [56,57]. Alkaline protease was applied to hydrolyzing the waste of shortfin scad (*Decapterus macrosoma*) [64]; after process optimization, the hydrolysate exhibited an ACE inhibition rate of 79.34 ± 0.79%, and the IC_50_ value of this hydrolysate was 3.98 mg/mL [63].

Most studies have adopted multiple proteases to hydrolyze fish proteins to screen for the optimal single enzyme, followed by process optimization to increase the ACE-inhibitory activity of the hydrolysates. In some cases, specific compound proteases are directly used for enzymatic hydrolysis. Up to now, microbial proteases have been the most commonly applied enzymes for aquatic protein hydrolysis due to their low cost and stable activity, and they significantly enhance the activity of fish-derived ACE-inhibitory peptides. Skipjack tuna (*Katsuwonus pelamis*) dark muscle was hydrolyzed with pepsin, papain, neutral protease, alkaline protease, and trypsin, separately, and neutral protease hydrolysate exhibited the highest ACE inhibition rate of 66.79 ± 1.53% at 1.0 mg/mL [33]; after optimizing the process, inhibition rate rose to 70.96%. Neutral protease was selected as the optimal enzyme for Pacific saury protein hydrolysis from acid protease, papain, trypsin, and alkaline protease; after process optimization, neutral protease hydrolysate achieved an ACE inhibition rate of 64.0%, at 1.0 mg/mL, with a hydrolysis degree of 18.5% and over 60% of peptides below 800 Da [48]. Likewise, papain, trypsin, bromelain, chymotrypsin, neutral protease, acid protease, and alkaline protease were applied to hydrolyze lizardfish scale gelatin, and the neutral protease hydrolysate achieved the highest ACE-inhibitory activity, showing an inhibition rate of 86.0 ± 0.40% and a peptide yield of 10.8% [54]. In contrast, tawny puffer (*Takifugu flavidus*) skin was hydrolysed using neutral protease, alkaline protease, and pepsin, and alkaline protease hydrolysate exhibited the optimal activity, with an IC_50_ value of at 0.1 mg/mL [46]. Another study treated salmon processing waste with six different proteases, and hydrolysate generated by alkaline protease possessed the highest ACE-inhibitory activity [35].

In some cases, compound proteases may produce hydrolysates with higher ACE-inhibitory activity than single proteases. For example, alkaline protease, flavorzyme, neutral protease, papain, pepsin, and trypsin were used to hydrolyze hairtail protein, and Protamex compound protease was screened as the optimal enzyme, yielding a hydrolysate with an ACE inhibition rate of 80.43% [65]. Similarly, Protamex compound protease and neutral protease were adopted separately to hydrolyze cobia (*Rachycentron canadum*) skin, and two batches of extracts recorded IC_50_ values of 0.221 mg/mL and 0.291 mg/mL, respectively, indicating that compound protease hydrolysate showed higher ACE-inhibitory activity [47]. Furthermore, the dark muscle by-products of albacore tuna (*Thunnus alalunga*) were hydrolyzed with a specific compound protease, and, after process optimization, the ACE inhibition rate reached 98.33 ± 2.01% at 1 mg/mL [27]. In addition, hairtail protein hydrolysate prepared using compound protease achieved an ACE inhibition rate of 80.43% (IC_50_ = 0.33 mg/mL), and the hydrophobic amino acid content was 36.72 g/100 g [65].

Microbial proteases also exhibit excellent hydrolysis effects. The roe protein of skipjack tuna (*Katsuwonus pelamis*) was hydrolyzed with six proteases (neutral protease, alkaline protease, flavorzyme, papain, pepsin, and trypsin), and the hydrolysate from flavorzyme hydrolysis showed the highest ACE inhibition rate of 62.87 ± 1.98% [28]. In comparison, the hydrolysate activities of thermolysin, pepsin, trypsin, and chymotrypsin on dried skipjack tuna protein were evaluated, and the thermolysin hydrolysate exhibited high ACE-inhibitory activity (IC_50_ = 29 μg/mL), superior to the boiling-water extract (IC_50_ = 174 μg/mL), indicating that highly active skipjack tuna ACE-inhibitory peptides are mostly released from insoluble proteins [34]. Similarly, the myofibrillar protein of striped snakehead (*Channa striatus*) was hydrolyzed with thermolysin and proteinase K, and the thermolysin hydrolysate exhibited higher activity (IC_50_ = 0.033 mg/mL) [41]. The IC_50_ values of the peptides isolated from the flavorzyme hydrolysate of skipjack tuna (*Katsuwonus pelamis*) roe [28] and the neutral protease hydrolysate of skipjack tuna (*Katsuwonus pelamis*) dark muscle [33] had obviously higher IC_50_ values than thermolysin hydrolysate of dried skipjack tuna powder [34].

Animal protease possesses strong substrate cleavage specificity and has also extensively been applied in aquatic bioactive peptide production. Bigeye tuna (*Thunnus obesus*) skin protein was hydrolyzed with neutral protease, alkaline protease, flavorzyme, and pancreatin, and the pancreatin hydrolysate exhibited the highest ACE inhibition rate of 60.00 ± 6.70% [20]. Similarly, the hydrolysates of six proteases on Alaska pollock (*Gadus chalcogrammus*) skin were compared, and the trypsin hydrolysate showed the optimal activity: ACE inhibition rate was 68.48 ± 2.96%, and increased to 79.62% after process optimization [21,39].

Studies have also demonstrated that pepsin is more suitable for the hydrolysis of aquatic protein. The blood and meat of bigeye tuna (*Thunnus obesus*) were hydrolyzed with six proteases, and pepsin hydrolysate exhibited the optimal ACE-inhibitory activity [29]. Similarly, Pacific cod (*Gadus macrocephalus*) skin gelatin was hydrolyzed with six proteases, and the inhibition rate of the pepsin hydrolysate was as high as 90.8% [66]. Another study also confirmed that pepsin hydrolysate showed the optimal activity in the hydrolysis of tuna steak protein [32].

Considering the human digestive and absorption environment, hydrolysis with simulated in vitro gastrointestinal digestive enzymes is also applied for bioactive peptides production. For instance, the hydrolysis effects of various proteases and simulated in vitro gastrointestinal digestive enzymes (trypsin + pepsin) on the swim bladder protein of bighead croaker (*Miichthys miiuy*) were compared, and the hydrolysate obtained with simulated in vitro gastrointestinal digestive enzymes exhibited the highest inhibitory activity, with an ACE inhibition rate of 51.37 ± 2.06% [53]. The hydrolysate obtained with simulated in vitro gastrointestinal digestive enzymes exhibited higher activity, with an ACE inhibition rate of 51.37 ± 2.06%. The peptide recovery rate also needs to be taken into consideration when ACE-inhibitory peptides are produced. Protein peptides were prepared from bigeye tuna (*Thunnus obesus*) skin using neutral protease, alkaline protease, flavorzyme, and pancreatin; pancreatin hydrolysate exhibited the highest ACE inhibition rate, but its protein recovery rate (43.79 ± 1.30%) was significantly lower than that of neutral protease (60.89 ± 0.80%) [20]. This may be explained by the fact that alkaline protease, as a serine endopeptidase, exhibits higher proteolytic efficiency.

### 4.2. Dual-Enzyme Hydrolysis

Compared with single-enzyme hydrolysis, dual-enzyme combinations can fully utilize the specific cleavage sites of different proteases. Protein hydrolysis can be enhanced through simultaneous or sequential hydrolysis modes to expand the range of peptides’ molecular weight distribution and increase the ACE-inhibitory activity of the hydrolysates, exhibiting a significant synergistic effect.

#### 4.2.1. Simultaneous Dual-Enzyme Hydrolysis

Simultaneous dual-enzyme hydrolysis refers to the simultaneous addition of two proteases into the reaction system to catalyze protein hydrolysis in a synchronous manner. Hairtail meat was hydrolyzed with alkaline protease and trypsin in simultaneous dual-enzyme mode, and an ACE inhibition rate of 77.48 ± 1.03% was achieved after process optimization, which was higher than that of single-enzyme hydrolysis with alkaline protease (53.66 ± 0.57%) or trypsin (47.74 ± 2.85%) [52]. Similarly, alkaline protease and trypsin were applied for the synchronous hydrolysis of Alaska pollock (*Gadus chalcogrammus*) skin to prepare highly active collagen peptides [39]. Likewise, alkaline protease combined with neutral protease was conducted to hydrolyze meat proteins extracted from yellowfin tuna (*Thunnus albacares*), and hydrolysates with ACE-inhibitory activity were obtained [30].

#### 4.2.2. Sequential Dual-Enzyme Hydrolysis

Sequential dual-enzyme hydrolysis refers to the sequential addition of two proteases into the reaction system to carry out hydrolysis in a stepwise manner, where peptide cleavage is successfully achieved through the specific cleavage sites of different proteases. For example, collagenase and flavorzyme were adopted for the stepwise hydrolysis of collagen from Atlantic cod (*Gadus morhua*) skin, and the proportion of peptides (<1 kDa) in the hydrolysate reached 82%, indicating the effective enrichment of low-molecular-weight peptides and their enhanced ACE-inhibitory activity [23]. Similarly, channel catfish protein was hydrolyzed through stepwise hydrolysis with trypsin and alkaline protease, and the hydrolysate with a molecular weight less than 500 Da accounted for 98.23% [22]. Likewise, the stepwise dual-enzyme hydrolysis of anglerfish (*Lophius litulon*) swim bladder collagen with alkaline and neutral proteases yielded a hydrolysate with a significantly higher ACE inhibition rate of 53.22 ± 2.63% than single-enzyme treatment [8].

Plant-derived bromelain and neutral protease were used for the stepwise hydrolysis of Alaska pollock (*Theragra chalcograma*) skin protein, and two ACE-inhibitory peptides were purified [24]. Atlantic salmon (*Salmo salar*) skin was also hydrolyzed to obtain collagen peptides through stepwise hydrolysis with alkaline protease and papain [36]. In addition to experimental approaches, computer-simulated hydrolysis by simulated hydrolysis with trypsin and papain, combined with simulated in vitro gastrointestinal hydrolysis with trypsin and pepsin, were performed to jointly screen myosin-derived ACE-inhibitory peptides from large yellow croaker (*Larimichthys crocea*), and the activity of the obtained peptides was predicted by a QSAR model [43].

Dual-enzyme hydrolysis usually involves single-enzyme screening at the first stage, followed by dual-enzyme combination for stepwise hydrolysis after optimization. For examples, flavorzyme and collagenase were screened as the optimal enzyme combination; after dual-enzyme stepwise hydrolysis, the ACE inhibition rate of the hydrolysate of Alaska pollock skin rose from 45.98% to 65.44% [38]. Collagenase and flavorzyme were also selected for the stepwise hydrolysis of Atlantic cod skin, and, after optimization, IC_50_ value was reduced from 3.78 to 1.46 mg/mL, confirming the dual-enzyme synergistic effect and the significant influence of the two enzymes’ hydrolysis order on the activity of the final product [60]. Furthermore, neutral protease and alkaline protease were successfully screened to hydrolyze bluefin tuna (*Thunnus thynnus*) meat protein by dual-enzyme stepwise hydrolysis [31]. Also, the combination of neutral protease and alkaline protease was adopted for the stepwise hydrolysis of European pilchard (*Sardina pilchardus*) meat protein, and the IC_50_ value of the hydrolysate was 96.09 ± 9.59 μg/mL [40].

### 4.3. Multi-Enzyme Combined Stepwise Hydrolysis

In order to verify the in vivo digestive stability of peptides by mimicking the body’s complex digestive environment, multi-enzyme combined stepwise hydrolysis mode is also adopted. This approach usually involves enzymatic hydrolysis with commercial proteases as a first step, followed by hydrolysis with simulated in vitro gastrointestinal digestive enzymes. Cobia (*Rachycentron canadum*) skin hydrolysates were prepared using compound protease and neutral protease, exhibiting ACE-inhibitory IC_50_ values of 0.221 and 0.291 mg/mL, respectively, which increased to 0.304 and 0.384 mg/mL after simulated gastrointestinal digestion, whereas subsequent purification yielded two highly bioactive peptides of IWW and AWW with IC_50_ values determined as 0.51 and 9.40 μM, respectively [47]. Furthermore, the hydrolysate could reduce the systolic and diastolic blood pressure of spontaneously hypertensive rats (SHRs).

After an initial hydrolysis of salmon muscle protein with pepsin, a subsequent digestion with trypsin and chymotrypsin was performed, and both the hydrolysate and its purified fractions were demonstrated to reduce systolic blood pressure in SHRs [67]. In contrast, swim bladder protein from bighead croaker (*Miichthys miiuy*) was hydrolyzed with simulated gastrointestinal enzymes, and six peptides (550–600 Da) with IC_50_ values ranging from 0.37 to 4.06 mg/mL were produced, among which SPYGF (IC_50_ = 623.26 μM) and SHGEY (IC_50_ = 1468.75 μM) showed relatively high activities [53], indicating that ACE-inhibitory peptides prepared by the single hydrolysis of fish protein with simulated in vitro gastrointestinal digestive enzymes have very low activity. The corresponding in vitro and in vivo activity data for the peptides discussed above are comprehensively compiled in Table 2.

## 5. Separation and Purification of Marine Fish-Derived ACE-Inhibitory Peptides

To obtain single bioactive peptide fragments, the protein hydrolysate needs to be subjected to continuous and stepwise separation and purification, followed by identification and molecular docking technology to obtain individual peptides [13] (Figure 3). Up to now, the separation, purification and identification technologies for ACE-inhibitory peptides have been well-established [68]. The stepwise separation and purification of bioactive peptides are typically achieved through chromatographic techniques, including ion exchange, size exclusion, affinity chromatography, and RP-HPLC. Among them, size-exclusion chromatography and RP-HPLC are the most widely adopted among these techniques, with peptide separation and purification based largely on molecular size and hydrophobic interactions [13].

Ultrafiltration serves as an efficient technique for the separation and enrichment of ACE-inhibitory peptide fractions [70]. Ultrafiltration is the initial step in separation: it is important for the screening of bioactive peptides. Ultrafiltration membranes with molecular weight cut-offs (MWCOs) of 1, 3, 5 and 10 kDa are usually adopted for stepwise gradient separation to obtain the fraction demonstrating the highest ACE-inhibitory activity (Figure 3a, different molecular weights determine different retention times). The bioactive fraction is then further fractionated by gel size-exclusion chromatography on Sephadex G-25 or G-15 for fractionation (Figure 3b, screening of the optimal active components). The obtained high-bioactivity gel fractions are then purified by RP-HPLC to screen purified peptides. Finally, the peptides are identified using liquid chromatography–tandem mass spectrometry (LC–MS/MS) or mass spectrometry (MS). RP-HPLC separates peptides based on their hydrophilicity, whereby hydrophilic peptides are eluted earlier than their hydrophobic counterparts [71].

To achieve the desired purification effect, multiple rounds of purification are usually carried out. For example, the protein hydrolysate of hairtail (*Trichiurus lepturus*) was subjected to ultrafiltration and three rounds of RP-HPLC purification, yielding two highly bioactive ACE-inhibitory peptides of ANSEVAQWR (IC_50_ = 89.58 μM) and EALVSQLTR (IC_50_ = 91.48 μM) [49]. After continuous and stepwise separation and purification, low-molecular-weight peptides are gradually and effectively enriched. Similarly, Atlantic cod (*Gadus morhua*) skin collagen peptides were produced by enzymatic hydrolysis, in which the peptide (<1 kDa) proportion accounted for 82%, and the proportion of peptides < 500 Da increased to 85% after ultrafiltration, then further to 97% after Sephadex G-25 gel filtration [23].

Ultrafiltration can be selectively performed using ultrafiltration membranes with different MWCO sizes, and the proportion of peptide fractions < 3 kDa in hydrolysate is generally high. For example, the proportion of <3 kDa peptide fraction in the hydrolysate of bigeye tuna (*Thunnus obesus*) skin reached 60%, with an ACE inhibition rate of 60.00 ± 6.70% [20]. Therefore, ultrafiltration membranes with a MWCO of 3 kDa are commonly used for ultrafiltration, and the obtained < 3 kDa fraction usually exhibits the highest activity, as reported on albacore tuna [27], bluefin tuna [31], Atlantic cod [39] and chub mackerel [50].

In addition, the content of peptide fractions < 1 kDa in the hydrolysate is also relatively high. For instance, the proportion of <1 kDa peptide fraction in the hydrolysate of Alaska pollock skin reached 93.08%, with 70.74% of peptides < 0.5 kDa, averaging 457.5 Da in molecular weight, with an ACE inhibition rate of 68.48 ± 2.96% [21]. Thus, ultrafiltration membranes with an MWCO of 1 kDa can also be used to obtain fractions with high activity. For example, two highly bioactive peptides of LGPLGHQ (IC_50_ = 4.22 μM) and MVGSAPGVL (IC_50_ = 3.09 μM) were isolated and purified from the <1 kDa ultrafiltration fraction of ocellate spot skate (*Okamejei kenojei*) skin gelatin hydrolysate [72]. Similar observations have been made on skipjack tuna [28], Alaska pollock skin [24], Pacific saury [48], and tawny puffer [46]. However, a highly bioactive ACE-inhibitory peptide (IC_50_ = 11.28 μM) was isolated and purified from the 1–5 kDa ultrafiltration fraction of tuna steak protein hydrolysate [32]. In addition, the more highly bioactive 3–5 kDa fraction (separated components) was obtained from striped snakehead (*Channa striatus*) myofibrillar protein hydrolysates, from which two highly bioactive peptides of VPAAPPK (IC_50_ = 0.45 μM) and NGTWFEPP (IC_50_ = 0.63 μM) were purified [41]. These results indicate that ultrafiltration membranes with high MWCO sizes are conducive to obtaining large-molecular-weight peptides with multi-amino acid sequences.

Generally, the ACE-inhibitory activity of the hydrolysate is continuously improved after ultrafiltration. However, some studies have also shown that the activity of the obtained fractions decreased after ultrafiltration. For example, <3 kDa ultrafiltration fraction of albacore tuna dark muscle hydrolysates showed a lower ACE inhibition rate (68.91% ± 1.06%) than the original hydrolysate (98.33% ± 2.01%), but subsequent affinity adsorption purified the potent peptide FPPDVA with an inhibition rate of 98.39% ± 0.61% and an IC_50_ value of 87.11 ± 1.02 μM [27]. Similarly, the <1 kDa ultrafiltration fraction of skipjack tuna roe protein hydrolysates showed a decreased ACE inhibition rate of 46.27% ± 2.49% compared with the original value (62.87% ± 1.98%), but the final peptides exhibited low activity (IC_50_ > 900 μM) after further purification, indicating that ultrafiltration alone is insufficient for enriching highly active peptides [28]. These findings indicate that ultrafiltration is mainly a preliminary separation step, and the activity of ultrafiltered fractions can be further improved through multi-stage ultrafiltration with different MWCO membranes.

Even when stepwise membrane separation is adopted, the phenomenon in which the activity of the fractions is lower than that of the original hydrolysate still occurs. For example, using 3.5, 5, and 10 kDa MWCO membranes for the stepwise ultrafiltration of skipjack tuna (*Katsuwonus pelamis*) dark muscle hydrolysate led to the isolation of two peptides of MKKS (IC_50_ = 546.04 μM) and MWN (IC_50_ = 729.65 μM), showing low activity [33]. This may be related to the large MWCO of the final step ultrafiltration membrane (3.5 kDa). However, sequential ultrafiltration effectively enriched ACE-inhibitory peptides from trypsin hydrolysates of Atlantic salmon bone proteins, with the <0.65 kDa fraction showing the highest activity (IC_50_ = 0.093 ± 0.004 μg/mL), whereas RP-HPLC purification process reduced the activity [25]. This may be due to the <0.65 kDa fraction being a coexisting system of multiple small-molecule bioactive peptides, which exerts a more significant synergistic inhibitory effect on ACE than the purified single-peptide fragment itself. This is because mixed peptides can form more hydrogen bonds and bind to the ACE active site to generate more stable complexes, thereby achieving a synergistic inhibitory effect [26]. Finally, a novel peptide FCLYELAR was obtained and showed a high ACE-inhibitory activity of IC_50_ = 31.63 μM) after synthesis.

Similarly, gradient ultrafiltration was performed on the skin hydrolysate of Alaska pollock (*Gadus chalcogrammus*) using membranes with MWCO sizes of 0.2, 1, 3, 5, and 10 kDa, and the <1 kDa fraction showed the highest ACE-inhibitory activity, indicating that a small MWCO does not necessarily lead to a better separation effect for bioactive peptides [24]. In addition, some studies perform gel chromatographic separation without using ultrafiltration membranes. For example, the meat protein hydrolysate of yellowfin tuna (*Thunnus albacares*) was directly separated using Sephadex G-25 gel filtration chromatography, yielding two potent ACE-inhibitory peptides of STHPHF (IC_50_ = 42.40 ± 0.25 μM, 724.33 Da) and LTFSY (IC_50_ = 32.93 ± 0.24 μM, 629.31 Da) (purified peptide) [30].

In many cases, stepwise separation and purification progressively enhance both ACE-inhibitory activity and the purity of the final fractions, ultimately yielding single-peptide components. Sequential ultrafiltration, ion-exchange chromatography, gel filtration, and RP-HPLC increased the ACE inhibition rate of Spanish mackerel by-product hydrolysates from 45.6% to 87.6% [62]. Similarly, a report showed that Sephadex G-15 gel filtration chromatography increased the ACE inhibition rate of channel catfish protein hydrolysates by 48.2% [22]. Another study also showed that the ultrafiltration and Sephadex G-15 gel filtration chromatography progressively enhanced the ACE-inhibitory activity of Pacific saury protein hydrolysates and ultimately yielded the bioactive peptide LEPWR with IC_50_ value of 99.5 μM [48]. In addition, through ultrafiltration and subsequent Sephadex G-15 gel filtration, the IC_50_ value of shortfin scad protein hydrolysates decreased from 3.98 to 0.20 mg/mL, and ACE-inhibitory peptide of GVGPVPAA (817.96 Da) was identified [63].

Furthermore, Atlantic cod (*Gadus morhua*) skin collagen hydrolysates purified through ultrafiltration, Sephadex G-25 gel filtration chromatography and HPLC progressively enhanced ACE-inhibitory activity, and ultimately the potent peptide GPAGPSGP (IC_50_ = 179 ± 12 μM) was identified [23]. Similarly, two novel highly bioactive peptides, GSAGPAGPSGPRGP and LGDARNSPAPP, were isolated from Alaska pollock (*Theragra chalcograma*) skin hydrolysate through sequential ultrafiltration, Sephadex G-25 gel filtration chromatography and RP-HPLC, exhibiting ACE inhibition rates of 91.41% and 88.52% (IC_50_ = 0.166 mM and 0.177 mM, respectively) [24]. The peptide of GPAGPSGP with IC_50_ value of 179 ± 12 μM was isolated and purified from Atlantic cod (*Gadus morhua*) skin collagen peptides [23], showing moderate activity. It has the identical sequence to that of Alaska pollock (*Theragra chalcograma*) [38], and its activity is similar to that of the peptide GPLGVP (IC_50_ = 105.8 μM) isolated from the skin hydrolysate of Atlantic cod (*Gadus morhua*) [39], as well as the peptides GSAGPAGPSGPRGP (IC_50_ = 166 μM) and LGDARNSPAPP (IC_50_ = 177 μM) obtained from the skin hydrolysate of Alaska pollock [24]. The activity of collagen peptides from cod is more than 5 times higher than that of collagen peptides from other fish species, such as anglerfish-derived peptide SEGPK [8] and sardine-derived peptide FIGR [73]. However, from the <1 kDa fraction of Pacific cod (*Gadus macrocephalus*) skin gelatin, GASSGMPG (662 Da, IC_50_ = 6.9 μM) and LAYA (436 Da, IC_50_ = 14.5 μM), were obtained through stepwise ultrafiltration, gel filtration, and RP-HPLC, indicating extremely high ACE-inhibitory activity [66].

After continuous and stepwise separation and purification, the activity of the finally obtained fractions or peptide fragments may be extremely high or extremely low. For example, low-molecular-weight ACE-inhibitory peptides purified from skin collagen peptides of Atlantic salmon (*Salmo salar* L.) exhibited extremely low activity (IC_50_ > 2500 μM) [36], and its activity was far lower than that of the three ACE-inhibitory peptides of FNVPLYE (IC_50_ = 7.72 μM), VWDPPKFD (IC_50_ = 9.10 μM) and FEDYVPLSCF (IC_50_ = 10.77 μM) gained from the protein hydrolysate of Atlantic salmon by-products [35]. Protein hydrolysate from European pilchard (*Sardina pilchardus*) showed ACE-inhibitory activity with an IC_50_ value of 96.09 ± 9.59 μg/mL [40]; the activity was higher than that of hydrolysates from Japanese pilchard (*Sardinops melanostictus*) (IC_50_ = 0.26 mg/mL) [74] and round sardinella (*Sardinella aurita*) (IC_50_ = 0.16 mg/mL) [75]. However, FIGR, FILR, FQRL, FRAL, KFL and KLF, screened from the European pilchard hydrolysate, had IC_50_ values ranging from 0.60 ± 0.01 mg/mL to 1.54 ± 0.14 mg/mL, corresponding to molar concentrations of 1261.72 ± 21.03 μM to 2886.00 ± 262.36 μM, exhibiting extremely low ACE-inhibitory activity [40].

In the process of peptide separation and purification, the bioactivity of crude fractions is commonly improved through progressive isolation in the early purification stages. However, the final purified peptides sometimes exhibit considerably low activity. For example, the stepwise purification of *Lophius litulon* swim bladder hydrolysates increased ACE-inhibitory activity from 53.22 ± 2.63% in the crude hydrolysate to 76.81 ± 2.76%, yielding three peptides (SEGPK, FDGPY, and SPGPW) with ACE inhibition rates of approximately 85% but relatively low activities (IC_50_ values of 1219.7, 1573.0, and 1308.5 μM, respectively) [8]. Conversely, the activity of crude fractions may gradually decrease during continuous separation and purification, while highly bioactive peptides can still be obtained in the final purification step. Taking *Takifugu flavidus* skin hydrolysates, for example, ultrafiltration increased the IC_50_ value from 0.10 to 0.58 mg/mL; after subsequent purification, the ACE inhibition rate reached 90% and yielded a novel octapeptide PPLLFAAL with an IC_50_ value of 28 μM [46].

Studies have also demonstrated that inconsistencies occur between ACE inhibition rate and IC_50_ values after separation and purification processes, indicating that a comprehensive evaluation of peptide bioactivity is indispensable rather than relying on a single indicator. The sequential ultrafiltration and purification of *Decapterus macrosoma* processing waste hydrolysates increased the ACE inhibition rate from 79.34 ± 0.79% slightly to 81.47 ± 0.41%, but reducing IC values from 3.98 to 1.59 mg/mL, and further cellular studies are needed to validate their ACE-inhibitory activity [63]. In addition to in vitro assays, further in vivo experiments are essential to comprehensively assess the ACE-inhibitory activity of purified peptides. For example, the ultrafiltration of *Miichthys miiuy* swim bladder hydrolysates increased the inhibition rate from 51.37 ± 2.06% to 62.9 ± 2.39%; Sephadex G-25 further increased the inhibition rate to 76.25 ± 3.61%. After further RP-HPLC purification, IC_50_ values of the obtained seven peptides ranged from 0.37 ± 0.06 mg/mL to 4.06 ± 0.37 mg/mL, among which SPYGF (IC_50_ = 623.26 μM) and SHGEY (IC_50_ = 1468.75 μM), with the lowest IC_50_ values, showed very low ACE-inhibitory activities [53]. However, all the seven peptides could significantly upregulate nitric oxide level and downregulate endothelin-1 secretion in human umbilical vein endothelial cells (HUVECs). Additionally, in vivo animal assays are essential to further verify the hypotensive effects of bioactive peptides. To date, in vivo validation has been conducted on ACE-inhibitory peptides derived from several fish species, including *Rachycentron canadum* skin [47], *Cololabis saira* [48], *Trichiurus lepturus* [76], *Saurida elongata* [54], and *Theragra chalcograma* skin [24]. However, relevant in vivo verification remains unreported for the majority of fish-derived ACE-inhibitory peptides.

After peptide identification by mass spectrometry, molecular docking is employed to characterize the binding mode and interaction energy between purified peptides and ACE (Figure 3c). The three-dimensional structures of identified peptides are constructed and energy-minimized using molecular mechanics. The crystal structure of human ACE (Protein Data Bank ID: 1O86) serves as the receptor. Docking simulations are performed with AutoDock Vina or Discovery Studio to predict binding poses, binding free energies (kcal/mol), and key intermolecular interactions, including hydrogen bonds, hydrophobic contacts, electrostatic interactions, and coordination with the catalytic Zn^2+^ cofactor at the ACE active site. As summarized in Table 2, molecular docking has been conducted for 23 of the 31 entries, with studies employing complementary approaches such as quantitative structure–activity relationship (QSAR) modeling [43,50], network pharmacology [38], and surface plasmon resonance (SPR) [49] to validate and extend docking predictions. The mechanistic insights derived from these docking studies are discussed in detail in Section 7.

Beyond chemical ACE inhibition assays, functional evaluation at the organ and whole-animal levels is essential to assess the antihypertensive potential of bioactive peptides (Figure 3d). Ex vivo vasorelaxation is typically assessed using isolated rat aortic ring preparations pre-contracted with phenylephrine (PE) or norepinephrine (NE). Endothelium-dependent and -independent mechanisms are distinguished by comparing endothelium-intact (+Endo) and endothelium-denuded (−Endo) rings. Pharmacological pathway dissection with Nω-nitro-L-arginine methyl ester (L-NAME, an endothelial NO synthase inhibitor) and indomethacin (a cyclooxygenase inhibitor) probes the involvement of the NO and prostaglandin pathways, respectively. For in vivo antihypertensive evaluation, the spontaneously hypertensive rat (SHR) model is widely employed. Peptides are administered by oral gavage at doses of 1–100 mg/kg body weight, and systolic blood pressure (SBP) and diastolic blood pressure (DBP) are monitored over 0–24 h by tail-cuff plethysmography or implantable telemetry. As catalogued in Table 2, in vivo SHR validation has so far been reported for only a limited number of peptides. Among them, the cobia skin-derived peptides IWW and AWW (IC_50_ = 0.51 and 9.40 μM) significantly reduced SBP and DBP in SHR [47], demonstrating that potent in vitro ACE inhibition can translate into measurable in vivo antihypertensive effects. Beyond SHR studies, cellular evidence from HUVEC assays further supports the vascular relevance of these peptides: peptides from bighead croaker (*Miichthys miiuy*) swim bladder hydrolysates significantly upregulated NO and downregulated endothelin-1 (ET-1) secretion [53], while peptides from skipjack tuna (*Katsuwonus pelamis*) roe and dark muscle provided cytoprotection against H_2_O_2_-induced HUVEC injury [28,33] (Table 2).

It should be emphasized that the materials reviewed here differ fundamentally in their level of structural characterization. Crude hydrolysates and partially purified fractions provide preliminary activity data but only limited structural insight, because their composite IC_50_ values may reflect synergistic or antagonistic interactions among numerous peptides; in contrast, sequence-identified purified peptides enable definitive structure–activity relationship analysis, molecular docking, and mechanistic investigation. Throughout this review, and in Table 1 and Table 2, each material is therefore classified as a crude hydrolysate, a separated fraction, or a purified peptide, and the conclusions are drawn at the level of evidence that each material can support.

## 6. Unique Characteristics of Marine Fish-Derived ACE-Inhibitory Peptides

ACE-inhibitory peptides have been widely reported from diverse protein sources, including terrestrial animal proteins (e.g., milk casein, whey, egg, pork, chicken), plant proteins (e.g., soybean, rice, pea, corn), and other marine organisms (e.g., mollusks, crustaceans, algae) [10,11]. However, marine fish-derived ACE-inhibitory peptides exhibit distinct structural and functional advantages, and there are several unique features regarding their sequence, physicochemical properties, and structure–activity relationships that differentiate them from their terrestrial and non-fish marine counterparts, including a generally higher ACE-inhibitory activity than that of peptides derived from terrestrial food sources (i.e., milk, chicken muscle and bovine) [77,78]. These characteristics are discussed below.

First, regarding amino acid composition, marine fish-derived ACE-inhibitory peptides are notably enriched in hydrophobic amino acids, particularly proline (P), leucine (L), isoleucine (I), valine (V), phenylalanine (F), tryptophan (W), and tyrosine (Y). These hydrophobic residues are known to be critical for binding to the S1, S1′, and S2′ subsites of the ACE active site, which are predominantly hydrophobic pockets [14]. The C-terminal tripeptide sequence has been recognized as the most critical determinant of ACE-inhibitory potency, with hydrophobic or aromatic residues (especially Pro, Phe, Trp, Tyr) at the C-terminal position strongly favoring ACE inhibition. A survey of the peptides reviewed herein reveals that a remarkably high proportion of potent marine fish-derived ACE-inhibitory peptides (IC_50_ < 100 μM) contain at least one aromatic residue (F, W, Y) or proline, aligning with the structural preferences of the ACE catalytic site. For instance, peptides such as FPPDVA, IWHHT, IWW, VPAAPPK, and IPYADFK all feature aromatic or proline residues at or near their termini. In contrast, ACE-inhibitory peptides from terrestrial plant sources (e.g., soybean, rice) often contain higher proportions of hydrophilic and charged residues (Asp, Glu, Arg, Lys), which may contribute to lower binding affinity for the hydrophobic ACE active pocket.

Second, marine fish proteins, particularly from the dark muscle of pelagic species (e.g., tuna, mackerel), contain higher proportions of myofibrillar proteins (myosin, actin, tropomyosin) and sarcoplasmic proteins compared to terrestrial muscle, which influences the peptide profiles released upon enzymatic hydrolysis. Notably, peptides derived from the myosin and actin of marine fish consistently exhibit higher ACE-inhibitory activity than those from collagen, which is the predominant protein in skin and scale hydrolysates. This observation is consistent with the fact that myofibrillar proteins contain a higher density of hydrophobic amino acid clusters that are preferentially cleaved by common proteases to release bioactive fragments. For example, thermolysin hydrolysates of skipjack tuna actin yielded peptides with IC_50_ values as low as 5.1 μM (IWHHT), whereas collagen-derived peptides from Atlantic salmon skin showed IC_50_ values above 2500 μM [34,36].

Third, marine fish-derived peptides are predominantly short-chain peptides (2–8 amino acid residues, molecular weight < 1000 Da), which is advantageous for oral bioavailability. Short-chain peptides are more resistant to gastrointestinal proteolysis and can be more efficiently absorbed across the intestinal epithelium via peptide transporters (PepT1) compared to longer peptides [77,79]. In contrast, peptides derived from terrestrial plant proteins often require more extensive hydrolysis to achieve comparable chain lengths, as plant storage proteins (e.g., glycinin, β-conglycinin) have more compact tertiary structures that limit protease accessibility. Furthermore, marine fish proteins generally have lower levels of cross-linking and post-translational modifications compared to terrestrial mammalian collagens, facilitating the more efficient enzymatic release of bioactive peptides.

Fourth, the wide diversity of marine fish species—encompassing over 30,000 known teleost species with vastly different habitats (tropical to polar, surface to deep-sea)—provides an unparalleled natural library of protein sequences for bioactive peptide discovery. This biodiversity translates into a broader spectrum of potential peptide sequences compared to the relatively limited number of terrestrial livestock and crop species commonly used for bioactive peptide production. Cold-water species such as Atlantic cod (*Gadus morhua*) and Alaska pollock (*Gadus chalcogrammus*) produce collagen with unique amino acid substitution patterns adapted to low-temperature environments (e.g., higher proline hydroxylation), which may yield structurally distinct peptide fragments upon hydrolysis. Moreover, the unusual amino acid compositions of certain marine fish tissues—for example, the high taurine content in the dark muscle of migratory pelagic species—may contribute to the multifunctional bioactivities observed in their hydrolysates.

## 7. Inhibition Mechanisms of Marine Fish-Derived ACE-Inhibitory Peptides

The mechanism by which a peptide inhibits ACE is fundamentally important for understanding its biological significance and therapeutic potential. ACE inhibitors can be classified into four major types based on enzyme kinetics: competitive inhibition, where the inhibitor competes with the substrate for binding to the active site; non-competitive inhibition, where the inhibitor binds to a site distinct from the active site and does not affect substrate binding; uncompetitive inhibition, where the inhibitor binds exclusively to the enzyme–substrate complex; and mixed-type inhibition, which displays characteristics of both competitive and non-competitive modes. The inhibition mode is typically determined through Lineweaver–Burk double reciprocal plot analysis by measuring ACE activity at varying substrate (HHL) and inhibitor concentrations. The inhibition modes reported for marine-derived ACE-inhibitory peptides, together with their potential applications, have been summarized in a recent review [80].

Among the marine fish-derived ACE-inhibitory peptides reviewed herein for which inhibition kinetics have been characterized, competitive inhibition is the most commonly reported mechanism. Taken together, fish-derived peptides have been reported to act as competitive [30,40,43,61,63], non-competitive [39,43,44,46], and mixed-type [23,24,31,38,48,50] inhibitors. The peptide GPAGPSGP from Atlantic cod (*Gadus morhua*) and Alaska pollock (*Gadus chalcogrammus*) skin collagen was also reported as a competitive inhibitor, binding to the S1 and S2 subsites of ACE [23,38]. LTFSY from yellowfin tuna (*Thunnus albacares*) muscle and RGVGPVPAA from shortfin scad (*Decapterus macrosoma*) by-products likewise exhibited competitive inhibition in kinetic analyses [30,63].

Non-competitive and mixed-type inhibition have also been reported for several marine fish-derived peptides. The peptide FPPDVA (IC_50_ = 87.1 μM) from albacore tuna (*Thunnus alalunga*) dark muscle was characterized as a non-competitive inhibitor, suggesting that it binds to a regulatory site distinct from the catalytic center while still effectively suppressing ACE activity [27]. The heptapeptide VPAAPPK (IC_50_ = 0.45 μM) from striped snakehead (*Channa striatus*) myofibrillar protein demonstrated mixed-type inhibition, indicating multiple binding modes that may contribute to its exceptionally high potency [41]. The peptides GSAGPAGPSGPRGP and LGDARNSPAPP from Alaska pollock (*Theragra chalcograma*) skin displayed mixed-competitive inhibition patterns, interacting with both the active pocket and the entrance channel of ACE [24]. The peptide LTGCP from bluefin tuna (*Thunnus thynnus*) muscle also exhibited mixed-type inhibition, whereas the inhibition mode of YPKP from the same source was not determined in that study; molecular docking analysis showed that these peptides occupy the ACE active site pocket through hydrogen bonds and hydrophobic interactions with key catalytic residues (His383, His387, Glu411) and the Zn^2+^ cofactor [31]. The peptide GPLGVP (IC_50_ = 105.8 μM) from Alaska pollock skin exhibited non-competitive inhibition kinetics as determined by Lineweaver–Burk plot analysis [39]. For peptides isolated from bighead croaker (*Miichthys miiuy*) swim bladders, non-competitive inhibition modes were observed, and the peptides were found to interact with key non-catalytic residues of ACE, stabilizing an inactive conformational state [53]. Recently, the pentapeptide RVCLP from lizardfish (*Saurida elongata*) muscle was likewise characterized as a non-competitive inhibitor that binds to Ala318, His347, Trp321, and Phe335 of ACE [58], whereas APFLAG from chub mackerel muscle hydrolysate displayed mixed-type inhibition [51]. Similarly, PLITT (IC_50_ = 48.73 ± 7.59 μM), purified from chub mackerel (*Scomber japonicus*) muscle hydrolysate, was characterized as a mixed-type inhibitor through QSAR-based screening, molecular docking and kinetic analysis [50]. Many terrestrial peptides act as competitive inhibitors, while some marine peptides exhibit mixed-type inhibition involving structural specificity and multi-site engagement, which highlights two dominant marine peptide strategies: hydrophobic pocket insertion and multi-point hydrogen bonding [81]. In addition, fish-derived peptides are more inclined to exhibit antihypertensive activity through multi-target, multi-mechanistic synergies [81].

The inhibition mechanism has important implications for in vivo efficacy. Competitive inhibitors may be more susceptible to displacement by accumulating endogenous substrate (angiotensin I) under pathological conditions with elevated renin–angiotensin system activity. In contrast, non-competitive and mixed-type inhibitors maintain their inhibitory effect regardless of substrate concentration, potentially offering more robust blood pressure-lowering effects in vivo. However, systematic studies directly comparing the in vivo antihypertensive efficacy of peptides with different inhibition mechanisms are currently lacking and represent an important direction for future research. Furthermore, the relationship between peptide sequence, molecular size, and inhibition mechanism merits deeper investigation: smaller peptides (di- and tripeptides) tend to act as competitive inhibitors by directly occupying the active site, while larger peptides may adopt non-competitive or mixed modes by binding to exosites and inducing conformational changes. A comprehensive understanding of inhibition mechanisms, combined with structural biology and computational approaches, will guide the rational design of next-generation ACE-inhibitory peptides with optimized potency, selectivity, and in vivo stability.

The antihypertensive effects of ACE-inhibitory peptides observed in SHR (Table 2, Figure 3d) are mediated through two interconnected physiological pathways. First, ACE catalyzes the conversion of angiotensin I (Ang I) to angiotensin II (Ang II), a potent vasoconstrictor that also stimulates aldosterone secretion and renal sodium retention via the AT1 receptor. By inhibiting ACE, bioactive peptides suppress Ang II production, thereby reducing peripheral vascular resistance and blood volume—the core mechanism exploited by all clinical ACE inhibitors [3,4]. Second, ACE is identical to kininase II, which degrades the vasodilator bradykinin into inactive fragments. ACE inhibition prolongs bradykinin half-life, which in turn activates endothelial bradykinin B2 receptors, stimulates endothelial NO synthase (eNOS) to produce nitric oxide (NO), and triggers cyclic guanosine monophosphate (cGMP)-mediated vascular smooth muscle relaxation [4]. This dual suppression of Ang II-mediated vasoconstriction and the enhancement of bradykinin-NO-mediated vasodilation accounts for the vasorelaxation effects depicted in the left panel of Figure 3d.

The pharmacological use of L-NAME and indomethacin as pathway probes in aortic ring assays serves to distinguish the relative contributions of NO and prostaglandin signaling to the observed vasorelaxation. L-NAME, by inhibiting eNOS, blocks NO production; if vasorelaxation is attenuated by L-NAME, this confirms NO pathway involvement. Indomethacin, by inhibiting COX, blocks prostaglandin synthesis; attenuation by indomethacin implicates prostaglandin-mediated mechanisms. Among the studies reviewed here, peptides from bighead croaker (*Miichthys miiuy*) swim bladder hydrolysates provided the most direct cellular evidence for NO-mediated endothelial effects, with all seven isolated peptides significantly upregulating NO and downregulating ET-1 in HUVECs [53]. However, systematic ex vivo vasorelaxation studies with pharmacological dissection remain largely absent for the majority of marine fish-derived peptides, representing a notable gap in the current characterization pipeline.

The SHR model bridges in vitro ACE inhibition and the complex physiological regulation of blood pressure in vivo. Among the five entries with SHR validation in Table 2, the cobia skin-derived tripeptide IWW (IC_50_ = 0.51 μM) [47] illustrates the feasibility of the full bench-to-animal translational trajectory: enzymatic hydrolysis → purification → sequence identification → in vitro IC_50_ determination → SHR oral administration → SBP/DBP reduction. However, it is important to note that in vivo efficacy is not solely determined by ACE-inhibitory potency. Gastrointestinal stability, intestinal absorption efficiency, first-pass metabolism, plasma protein binding, and the compensatory activation of alternative pressor systems all modulate the ultimate blood pressure response. Competitive ACE inhibitors, which constitute the majority of characterized marine fish-derived peptides (Section 7), may be particularly susceptible to displacement by elevated endogenous Ang I levels under hypertensive conditions, potentially limiting their in vivo duration of action.

While a substantial number of marine fish-derived peptides exhibit potent in vitro ACE-inhibitory activity—with 23 out of the 31 entries compiled in Table 2 supported by molecular docking and 8 by cell-based assays—only 5 have undergone in vivo validation in spontaneously hypertensive rats (SHRs), and none has progressed to human clinical trials, although marine-derived peptides as a class have been reported to inhibit ACE activity and to control blood pressure in both in vitro and in vivo preclinical models as well as in human studies [82]. This pronounced translational gap reflects several well-recognized barriers: susceptibility to gastrointestinal proteolysis, limited intestinal absorption of intact peptides, rapid plasma clearance, and the complexity of blood pressure regulation involving multiple compensatory pathways beyond ACE inhibition. Consequently, low in vitro IC_50_ values, even when corroborated by favorable docking scores, do not reliably predict in vivo antihypertensive efficacy. Recent studies have begun to address these barriers by integrating inhibition kinetics with intestinal transport assays and network-pharmacology analysis; for example, two novel ACE-inhibitory peptides from yellowfin tuna muscle were characterized in terms of their inhibitory mechanism, transport route, and network-pharmacology profile [83]. By explicitly cataloging the biological validation status of each entry—whether supported by cellular assays, molecular docking, or SHR studies—Table 2 enables readers to assess the translational maturity of individual peptides at a glance. This stratified evidence framework serves as a practical guide for prioritizing candidates with multi-level validation for further preclinical and clinical development, and highlights the critical need for integrated in vitro–in vivo evaluation pipelines in future marine bioactive peptide research.

## 8. Future Prospects

First of all, future work should aim for strengthening the comprehensive evaluation of ACE-inhibitory peptides through combined in vitro and in vivo bioactivity assessments. At present, the bioactivity evaluation of ACE-inhibitory peptides predominantly relies on conventional in vitro chemical analytical approaches, particularly the hippuric acid high-performance liquid chromatography (HHL-HPLC) method. This technique can directly obtain key indicators, including ACE inhibition rate, IC value, and gastrointestinal digestion stability, which has been established as the conventional method for the screening and preliminary verification of ACE-inhibitory peptide bioactivity. However, multi-dimensional evaluation via supplementary in vitro cellular assays and in vivo animal experiments.

In terms of in vitro evaluation, cellular-level bioactivity verification should be further supplemented on the basis of chemical activity tests. Multiple cell models, such as HUVECs and renal tubular cells, can be adopted to quantify the expression level and inhibitory efficiency of ACE, so as to further validate the bioactivity of candidate peptides. Nitric oxide (NO), as a critical vascular relaxation factor in the human body, exerts a vital vasodilatory and hypotensive effect and plays an indispensable role in maintaining cardiovascular homeostasis. Therefore, evaluating the regulatory effect of bioactive peptides on NO secretion and endothelial cell function can effectively supplement the mechanism of their antihypertensive activity. Furthermore, with the rapid development of bioinformatics and computational biology, computer simulation technology has become a crucial auxiliary tool for the screening and mechanistic study of ACE-inhibitory peptides. Characterized by high throughput, low cost, and strong predictive performance, this approach serves as an efficient pre-screening strategy to rapidly narrow down the scope of candidate peptides. Quantitative structure–activity relationship (QSAR) models can construct quantitative mathematical correlations between peptide molecular structural parameters and bioactivity, enabling an accurate prediction of the IC values of ACE-inhibitory peptides without extensive in vitro experiments, which facilitates virtual screening and structure–activity relationship analysis of bioactive peptides. Considering the complexity and randomness of protein enzymatic hydrolysis, which hinder efficient peptide screening, the integration of computer simulation prediction and experimental verification can serve as a promising and efficient strategy for exploring novel ACE-inhibitory peptides.

In addition, in vivo animal experiments are the core link to verify the hypotensive efficacy and physiological function of bioactive peptides, as animal models can better reflect the comprehensive effects of peptides in gastrointestinal digestion, absorption, metabolic translation, and blood pressure regulation. Nevertheless, current studies on the in vivo functional verification of marine-derived ACE-inhibitory peptides remain inadequate. The spontaneously hypertensive rat (SHR) model, which possesses pathological characteristics highly consistent with human essential hypertension, is the most widely recognized animal model for evaluating the hypotensive effect of bioactive peptides. It is also a critical preclinical verification platform and an essential bridge between laboratory research and clinical application transformation. In future studies, systematic efficacy evaluation based on diverse animal models and human trials should be strengthened, which will further promote the industrial transformation of marine-derived ACE-inhibitory peptides in the fields of health food development and pharmaceutical research.

Secondly, more attention should be paid to the yield improvement of high-activity target fractions of marine fish-derived peptides. Most existing studies follow conventional technical routines, including enzyme screening, process optimization, ultrafiltration, gel filtration chromatography, RP-HPLC purification, and mass spectrometry identification. Although this classic technical route can obtain bioactive peptide monomers, insufficient optimization in each purification stage easily leads to low recovery efficiency of high-activity components. Therefore, future research needs to strengthen the targeted optimization and systematic exploration of each unit operation in the purification process. Optimizing separation parameters and purification strategies can effectively enhance the bioactivity of intermediate fractions and final peptide products, thereby significantly improving the yield of high-activity ACE-inhibitory peptide components and promoting efficient resource utilization.

Thirdly, it is essential to expand the preparation methodologies of marine fish-derived bioactive peptides. At present, the production of fish-derived ACE-inhibitory peptides overwhelmingly relies on single enzymatic hydrolysis technology, which limits the improvement of hydrolysis efficiency and product functional characteristics. Hence, novel auxiliary preparation methods should be explored and combined with traditional enzymatic hydrolysis to achieve synergistic optimization. For instance, microbial fermentation can effectively alleviate the bitter taste of enzymatic peptides, improve flavor and palatability, and optimize the functional characteristics of peptide products. Peptides with excellent sensory quality and health benefits possess broader application prospects in functional foods and nutraceutical products, which represents a vital development trend for marine peptide industrialization.

Fourthly, multi-functional screening and synergistic effect evaluation for fish-derived peptides should be strengthened. Currently, most existing studies have merely focused on the evaluation of single biological activities. Given the complex and diverse protein sequences of marine fish resources, bioactive peptides usually possess multiple potential functional characteristics. Future research should emphasize the excavation of dual- or multi-functional peptides with synergistic biological effects. Combined with multi-level in vitro and in vivo activity evaluation systems, the synergistic mechanism among different biological functions can be clarified. The exploration of multi-effect peptides can diversify the functional attributes of marine fish-derived peptides, expand their application scenarios, and provide diversified nutritional and healthy raw materials for different consumer groups.

Fifthly, full attention should be paid to the sustainable utilization of raw materials for marine fish-derived peptides. Marine fish resources are abundant and widely distributed, providing high-quality protein sources for bioactive peptide preparation. To improve resource utilization efficiency and economic added value, priority should be given to fish processing by-products as the raw materials for ACE-inhibitory peptide preparation. Efficiently utilizing waste by-products for high-value peptide development can reduce protein resource waste, realize the full utilization of aquatic processing resources, and achieve dual economic and environmental benefits.

Finally, accelerating the integration of emerging artificial intelligence (AI) techniques into ACE-inhibitory peptide research and development is therefore critical for industrial upgrading. In this regard, the integration of emerging AI techniques is highly recommended to advance the research and development of ACE-inhibitory peptides. Strategies such as peptidomics analysis and machine learning-assisted screening can be adopted for bioactive peptide mining. This prospective methodology can substantially improve screening efficiency and accuracy, while overcoming the drawbacks of traditional screening techniques, namely, time-consuming procedures and complicated operational processes.

## Figures and Tables

**Figure 1:**
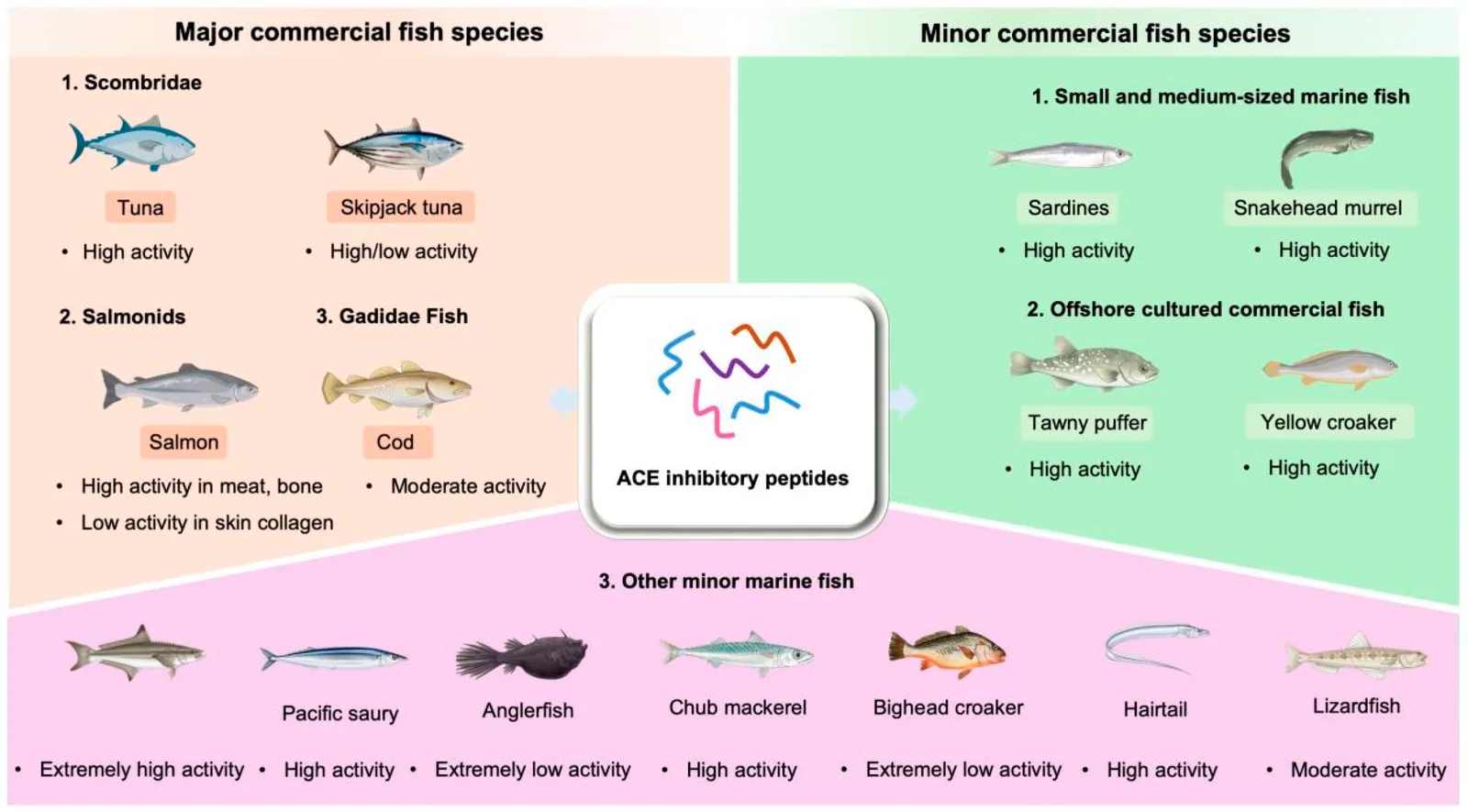
Classification of marine fish species used to produce ACE-inhibitory peptides.

**Figure 2:**
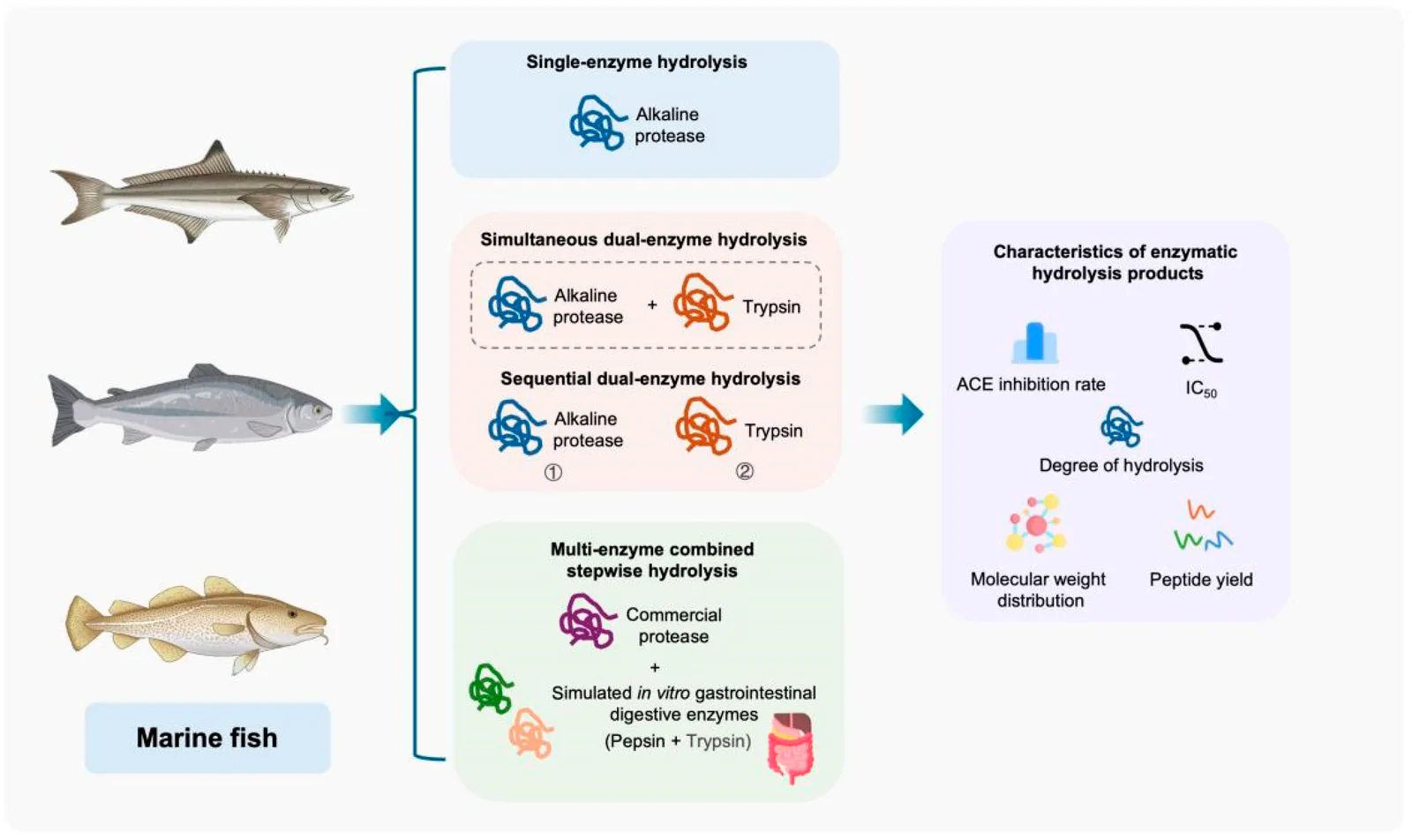
Enzymatic hydrolysis for preparing marine fish-derived ACE-inhibitory peptides.

**Figure 3:**
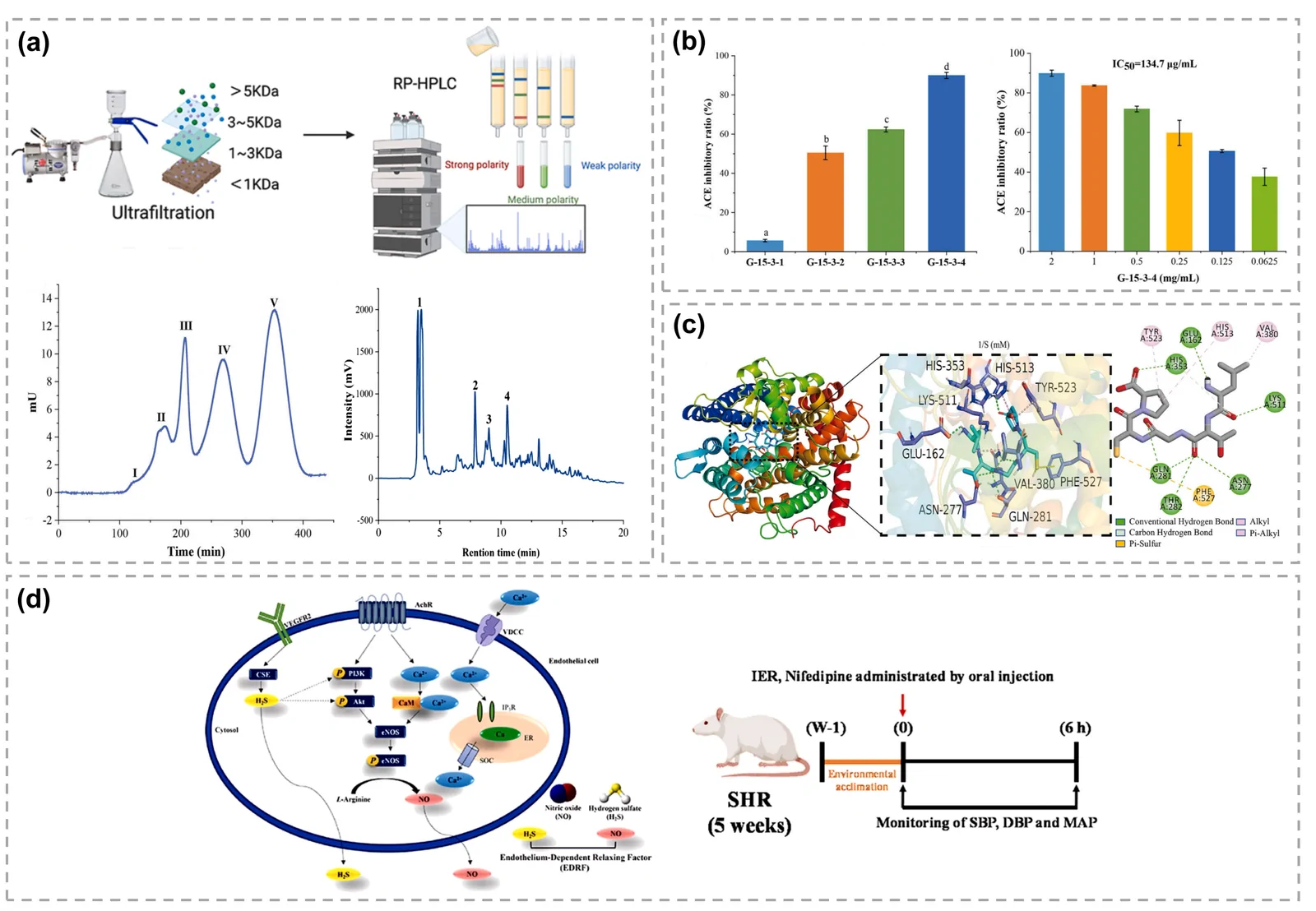
Overview of the experimental pipeline for marine fish-derived ACE-inhibitory peptide research [31,69]. (**a**) Separation and purification workflow: ultrafiltration (UF) → gel filtration chromatography (GFC) → reversed-phase high-performance liquid chromatography (RP-HPLC). (**b**) G-15 is a grade of Sephadex, a cross-linked dextran gel filtration medium (Cytiva, formerly GE Healthcare), used in gel filtration chromatography (GFC) for size-based fractionation. In vitro ACE-inhibitory activity determined by the hippuryl-histidyl-leucine high-performance liquid chromatography (HHL-HPLC) assay; IC_50_ values derived from dose–response curves. Data: mean ± standard deviation (SD), *n* ≥ 3. a, b, c and d on the figure indicated significant difference among data, *p* < 0.05. (**c**) Molecular docking of peptides with human angiotensin-converting enzyme (ACE) (Protein Data Bank (PDB) ID: 1O86) using autodock 4.2.6; key interactions include hydrogen bonds, hydrophobic contacts, and Zn^2+^ coordination. (**d**) Vasorelaxation assessed by rat aortic ring assay (±endothelium; L-NAME and indomethacin as pathway probes), and in vivo antihypertensive effects measured as MAP, SBP, and DBP in SHR following oral gavage (1–100 mg/kg). Data: mean ± SEM, n = 6–8. PE (phenylephrine), NE (norepinephrine), L-NAME (Nω-nitro-L-arginine methyl ester), eNOS (endothelial nitric oxide synthase), COX (cyclooxygenase), NO (nitric oxide), MAP (mean arterial blood pressure), SHR (spontaneously hypertensive rat), SBP (systolic blood pressure), DBP (diastolic blood pressure), SEM (standard error of the mean), ET-1 (endothelin-1), cGMP (cyclic guanosine monophosphate), Ang I (angiotensin I), Ang II (angiotensin II), AT1 (angiotensin II type 1 receptor).

**Table 1:** Enzymatic hydrolysis, separation, purification and identification methods of marine fish-derived ACE-inhibitory peptides.

Fish Species and Tissues	Enzymatic Hydrolysis Method	Separation and Purification Method	Identification Method	Hydrolysate/Fraction/Peptide Sequence	Molecular Weight (Da)	Reference
Dark muscle of albacore tuna (*Thunnus alalunga*)	Single enzyme—compound protease	Ultrafiltration (3, 5 and 10 kDa)/Affinity purification/Solid-phase synthesis	LC–MS/MS	Ultrafiltration fraction FPPDV KFPWGDGN	<3000 and stepwise 644.32 919.42	[27]
Skin of bigeye tuna (*Thunnus obesus*)	Single enzyme—trypsin	Ultrafiltration		Hydrolysate	<3000 (60%)	[20]
Muscle of yellowfin tuna (*Thunnus albacares*)	Simultaneous hydrolysis with alkaline and neutral protease	Sephadex G-25 gel filtration/HPLC/Solid-phase synthesis	LC–MS/MS	Gel fraction STHPHF LTFSY	742–182 (80%) 724.33 629.31	[30]
Muscle of bluefin tuna (*Thunnus thynnus*)	Dual enzyme—stepwise hydrolysis with neutral and alkaline protease	Ultrafiltration (3, 10 kDa)/Sephadex G-15 gel filtration/RP-HPLC/Solid-phase synthesis	Q-MS/MS	LTGCP YPKP	489.59 503.60	[31]
Skin of Atlantic salmon (*Salmo salar* L.)	Dual enzyme—stepwise hydrolysis with alkaline protease and papain	RP-HPLC	Q-TOF-MS/MS	GR RER GPR	202.21 459.24 328.19	[36]
Bone of Atlantic salmon (*Salmo salar*)	Single enzyme—trypsin hydrolysis	Ultrafiltration (0.65, 3, 5 and 10 kDa)/RP-HPLC	Q-TOF-LC–MS/MS	Ultrafiltration fraction CLYELAR	<650 1041.2	[25]
Dark muscle of skipjack tuna (*Katsuwonus pelamis*)	Single enzyme—neutral protease	Ultrafiltration (3.5, 5 and 10 kDa)/Sephadex G-25 gel filtration	ESI-Q-TOF-MS	Ultrafiltration fraction MKKS MWN LPRS MEKS	<3500 492.64 449.53 471.56 493.58	[33]
Roe of skipjack tuna (*Katsuwonus pelamis)*	Single enzyme—flavorzyme	Ultrafiltration (1, 3.5 and 5 kDa)/DEAE-52 ion-exchange/Sephadex G-25 gel filtration	-	WGESF IKSW YSHM WSPGF	624.63 532.63 536.58 592.63	[28]
Muscle of skipjack tuna (*Katsuwonus pelamis*)	Single enzyme—alcalase	Ultrafiltration (3.5, 5 and 10 kDa)/Sephadex G-25 gel filtration/RP-HPLC	ESI-Q-TOF-MS	SP VDRYF VHGVV YE FEM FWRV	202.3 698.9 509.7 310.4 425.6 606.8	[59]
Skin of Atlantic cod (*Gadus morhua*)	Dual enzyme—stepwise hydrolysis with collagenase and flavorzyme	Ultrafiltration (1, 3 and 10 kDa)/Sephadex G-25 gel filtration/Pre-HPLC/Solid-phase synthesis	UPLC-MS/MS	Hydrolysate Ultrafiltration fraction Gel fraction MLGP MWW GPAGPSGP	<1000 (82%) <500 (85%) <500 (97%) 516.54 521.63 638.67	[23]
Skin of Atlantic cod (*Gadus morhua*)	Dual enzyme—stepwise hydrolysis with hydrolytic protease and flavorzyme	Ultrafiltration (1, 3 and 10 kDa)	HPLC	Ultrafiltration fraction	<1000	[60]
Skin of Alaska pollock (*Gadus chalcogrammus*)	Dual enzyme—stepwise hydrolysis with flavorzyme and collagenase	Ultrafiltration (0.2 kDa–10 kDa)/Sephadex G-25 gel filtration chromatography/Pre-HPLC	HPLC–MS/MS	GPAGPSGP	663.67	[38]
Skin of Alaska pollock (*Theragra chalcograma*)	Dual enzyme—stepwise hydrolysis with bromelain and neutral protease	Ultrafiltration (1, 3, 5 and 10 kDa)/Sephadex G-25 gel filtration/RP-HPLC/Solid-phase synthesis/Computer simulation	LC–MS-MS/UHPLC/MALDI-TOF-MS	Ultrafiltration fraction Gel fraction GSAGPAGPSGPRGP (GP1) LGDARNSPAPP (GP2)	505 400–1708 1163.56 1093.55	[24]
Skin of Alaska pollock (*Gadus chalcogrammus*)	Dual enzyme—stepwise hydrolysis with alkaline protease and trypsin	Ultrafiltration/Sephadex G-25 gel filtration/RP-HPLC	HPLC–Q-TOF-MS	Ultrafiltration fraction GPLGVP VLYPVK VFLENVLR FEEF	<3000 538.31 692.68 945.04 570.38	[39]
Skin of Alaska pollock (*Gadus chalcogrammus*)	Single enzyme—trypsin	-	SDS-PAGE/HPLC	Hydrolysate	<1000 (93.08%) <500 (70.74%)	[21]
Muscle of lizardfish (*Saurida elongata*)	Single enzyme—neutral protease	Metal affinity-immobilized magnetic liposomes (MA-IML)/RP-HPLC	MALDI-TOF/TOF-MS	VYP	413.47	[57]
Scale gelatin from lizardfish (*Synodus macrops*)	Single enzyme—neutral protease	HiTrap™ Capto™ Q strong anion exchange Resin (Cytiva Life Sciences, Uppsala, Sweden)/Sephadex G15 gel filtration (self-made)/UNO Q1 strong anion exchange	Nano-LC–MS/MS	Hydrolysate AGPPGSDGQ PGAK	<2000 (93.2%) 1137.3	[54]
Muscle of European pilchard (*Sardina pilchardus*)	Dual enzyme—stepwise hydrolysis with neutral and alkaline protease	Ultrafiltration (3 and 5 kDa)/Solid-phase synthesis	LC–MS/MS	Ultrafiltration fraction FIGR FILR FQRL FRAL KFL KLF	<3000 475.54 517.62 533.61 489.57 377.48 377.48	[40]
Muscle of hairtail (*Trichiurus lepturus*)	Dual enzyme—synchronous hydrolysis with alkaline protease and trypsin	Ultrafiltration (10, 3 kDa)/Sephadex G-25 gel filtration	LC–MS/MS	Ultrafiltration fraction VELGV SVAPL SLAGLP TDIGVAGI LGGGPI	<3000 516.30 486.29 557.33 745.41 513.30	[52]
Muscle of hairtail (*Trichiurus lepturus*)	Single enzyme—trypsin	Ultrafiltration (3 kDa)/RP-HPLC	LC–MS/MS	ANSEVAQWR EALVSQLTR	1040.5 1014.5	[49]
Muscle of ribbonfish (*Trichiurus lepturus*)	Single enzyme—alkaline	Ultrafiltration (10, 3 kDa)/Sephadex G-25 gel filtration	LC–MS/MS	FAGDDAPR QGPIGPR GPTGPAGPR	847.97 723.93 809.01	[61]
Muscle of Pacific saury (*Cololabis saira*)	Single enzyme—neutral protease	Ultrafiltration (1 kDa)/Sephadex G-25 gel filtration/RP-HPLC	Nano-LC–QE-MS/MS	LEPWR TPAPVPTP VFPLK	681.34 706.35 566.34	[48]
Muscle of chub mackerel (*Scomber japonicus*)	Single enzyme—alkaline protease	Ultrafiltration (3 kDa)/Sephadex G-15 gel filtration/RP-HPLC	LC–MS/MS	PLITT LTPFT	543.63 564.66	[50]
By-products (muscle) of Spanish mackerel (*Scomberomorus niphonius*)	Single enzyme—alkaline protease	Ultrafiltration (3, 5 and 10 kDa)/DEAE-Sepharose FF anion-exchange/Sephacryl S-100 HR gel filtration (GE, Marlborough, MA, USA) /RP-HPLC	-	Ultrafiltration fraction	<3000	[62]
Muscle of longsnout catfish (*Leiocassis longirostris*)	Dual enzyme—stepwise hydrolysis with trypsin and alkaline protease	Ultrafiltration (3 kDa) (Millipore, Burlington, MA, USA)/DA201-C resin (Solarbio, Beijing, China)/Sephadex G-15 gel filtration (Solarbio, Beijing, China)	HPLC	Hydrolysate	<500 (98.2%)	[22]
Swim bladder of bighead croaker (*Miichthys miiuy*)	Dual enzyme—stepwise hydrolysis with in vitro simulated digestion (GI)	Ultrafiltration (1, 5 and 10 kDa)/Sephadex G-25 gel filtration/RP-HPLC	ESI-Q-TOF-MS	Ultrafiltration fraction SPYGF SHGEY VIGPF GPFGTD GPWGPA DEGPE EVGIQ	<1000 569.60 591.55 531.63 592.59 583.63 545.49 544.58	[53]
Skin of tawny puffer (*Takifugu flavidus*)	Single enzyme—alkaline protease	Ultrafiltration (1, 3, 5, 10, 50 kDa)/SP-HPLC/Sephadex G-15 gel filtration	LC–MS/MS	Hydrolysate PPLLFAAL	<1000 841.05	[46]
Swim bladder of monkfish (*Lophius litulon*)	Dual enzyme—stepwise hydrolysis with alkaline and neutral protease	Ultrafiltration (1, 3.5, 5 kDa)/Sephadex G-25 gel filtration/RP-HPLC	ESI-Q-TOF-MS	Ultrafiltration fraction SEGPK FDGPY SPGPW	<1000 516.5 597.6 542.6	[8]
Muscle of large yellow croaker (*Larimichthys crocea*)	Computer-simulated hydrolysis (trypsin + papain)/in vitro simulated digestion (GI)	Ultrafiltration (0.5, 1, 3, 5 kDa)/Solid-phase synthesis	LC–MS/MSQSAR	Ultrafiltration fraction YNL IPYADFK LYDHLGK INEMLDTK IHFGTTGK NWPWMK FYEPFM	<1000 408.45 852.97 844.95 963.11 859.98 861.04 832.97	[43]
Muscle of large yellow croaker (*Larimichthys crocea*)	Dual enzyme—stepwise hydrolysis with pepsin and trypsin	Computer simulation	Solid-phase synthesis	WAR WQR	431.52 488.58	[44]
By-products (bone, skin and tail) of shortfin scad (*Decapterus macrosoma*)	Single enzyme—alkaline protease	Ultrafiltration (3, 5 and 10 kDa)/Sephadex G-25 gel filtration/RP-HPLC	MALDI-TOF/TOF-MS	Ultrafiltration fraction Gel fraction RGVGPVPAA	<3000 1000–5000 822.85	[63]
Skin of cobia (*Rachycentron canadum*)	Stepwise hydrolysis with compound protease and in vitro gastrointestinal digestion (GI)	Sephadex G-25 gel filtration (Amersham Pharmacia Biotech AB, Uppsala, Sweden)/RP-HPLC (L-7100, Hitachi, Tokyo, Japan)/Solid-phase synthesis	Edman degradation/Protein sequencer	Gel fraction WAA AWW IWW WL	450–630 346.39 461.52 503.60 317.39	[47]

**Table 2:** In vitro and in vivo activity evaluation of marine fish-derived ACE-inhibitory peptides and their derivatives.

Fish Species and Tissues	Source Protein	Hydrolysate/Fraction/ Peptide	In Vitro Activity Inhibition Rate (R)/IC_50_ Value	Inhibition Mode	Availability of Cell-Based Assays and/or Molecular Docking/QSAR	Availability of Animal (SHRs) Experiment	Reference
Dark muscle of albacore tuna (*Thunnus alalunga*)	Myoglobin	Hydrolysate Ultrafiltration fraction Affinity adsorption fraction FPPDVA KFPWGDGN	R = 98.33 ± 2.01% R = 68.91 ± 1.06% R = 98.39 ± 0.61% IC_50_ = 87.11 ± 1.02 μM IC_50_ = 297.51 ± 0.95 μM	Non-competitive -	Molecular docking -	--	[27]
Skin of bigeye tuna (*Thunnus obesus*)	Collagen	Hydrolysate	R = 60.00 ± 6.70%	--	--	--	[20]
Muscle of yellowfin tuna (*Thunnus albacares*)	Myosin & actin	Gel fraction STHPHF LTFSY	R = 64.63 ± 0.58% IC_50_ = 30.71 ± 0.18 μg/mL (40.4 μM) IC_50_ = 20.72 ± 0.15 μg/mL (32.92 μM)	Mixed Competitive	Molecular docking +	--	[30]
Muscle of Atlantic tuna (*Thunnus thynnus*)	Myosin & actin	LTGCP YPKP	IC_50_ = 64.3 μM IC_50_ = 139.6 μM	Mixed -	Molecular docking +	--	[31]
Skin of Atlantic salmon (*Salmo salar* L.)	Collagen	GR RER GPR	IC_50_ = 0.73 ± 0.05 mg/mL (3158.53 μM) IC_50_ = 0.89 ± 0.06 mg/mL (1937.98 μM) IC_50_ = 1.05 ± 0.13 mg/mL (3199.37 μM)	--	Molecular docking	--	[36]
Bone of Atlantic salmon (*Salmo salar*)	Collagen	Hydrolysate Ultrafiltration fraction FCLYELAR	IC_50_ = 7.14 ± 0.05 μg/mL IC_50_ = 0.093 ± 0.004 μg/mL IC_50_ = 0.26 ± 0.02 μg/mL (31.63 μM ± 0.15 μM)	Non-competitive	Molecular docking	--	[25]
Dark muscle of skipjack tuna (*Katsuwonus pelamis*)	Myoglobin	Hydrolysate Ultrafiltration fraction Gel fraction MKKS MWN LPRS MEKS	R = 66.79 ± 1.53% R = 51.05 ± 2.14% R = 61.21 ± 1.93% IC_50_ = 0.269 ± 0.006 mg/mL (546.04 ± 12.18 μM) IC_50_ = 0.328 ± 0.035 mg/mL (729.65 ± 77.86 μM) IC_50_ = 0.495 ± 0.024 mg/mL (1049.71 ± 50.90 μM) IC_50_ = 0.527 ± 0.030 mg/mL (1067.71 ± 60.78 μM)	--	Cell-based assays	--	[33]
Roe of skipjack tuna (*Katsuwonus pelamis*)	Vitellogenin	Hydrolysate Ultrafiltration fraction DEAE-52 fraction Gel fraction WGESF IKSW YSHM WSPGF	R = 62.87 ± 1.98% R = 46.27 ± 2.49% R = 54.76 ± 2.87% R = 63.75 ± 3.06% IC_50_ = 0.93 ± 0.07 mg/mL (1488.88 ± 112.07 μM) IC_50_ = 0.79 ± 0.06 mg/mL (1483.21 ± 112.65 μM) IC_50_ = 0.49 ± 0.06 mg/mL (913.19 ± 111.82 μM) IC_50_ = 0.67 ± 0.05 mg/mL (1130.55 ± 84.37 μM)	--	Cell-based assays/ Molecular docking + + +	--	[28]
Muscle of skipjack tuna (*Katsuwonus pelamis*)		Hydrolysate Ultrafiltration fraction Sephadex G-25 SP VDRYF VHGVV YE FEM FWRV	72.71% 47.26 ± 0.64% 55.87 ± 2.15% IC_50_ = 0.06 ± 0.01 mg/mL IC_50_ = 0.28 ± 0.03 mg/mL IC_50_ = 0.90 ± 0.16 mg/mL IC_50_ = 0.80 ± 0.03 mg/mL IC_50_ = 2.18 ± 0.20 mg/mL IC_50_ = 0.76 ± 0.10 mg/mL	--	Molecular docking/ Cell-based assays + + + + +	--	[59]
Skin of Atlantic cod (*Gadus morhua*)	Collagen	Hydrolysate Ultrafiltration fraction Gel fraction RP-HPLC fraction MLGP MWW GPAGPSGP	IC_50_ = 1.46 ± 0.08 mg/mL, R = 65.44 ± 0.53% IC_50_ = 0.86 ± 0.03 mg/mL, R = 87.65 ± 0.24%, IC_50_ = 0.49 ± 0.06 mg/mL, R = 49.42 ± 0.61% IC_50_ = 0.28 ± 0.04 mg/mL, R = 57.55 ± 0.69% IC_50_ = 683.1 ± 17 μM IC_50_ = 497.6 ± 24 μM IC_50_ = 179.0 ± 12 μM	- - Mixed	- - Molecular docking		[23]
Skin of Alaska pollock (*Gadus chalcogrammus*)	Collagen	Hydrolysate Ultrafiltration fraction Gel fraction HPLC fraction GPAGPSGP MWW MLGP	R = 65.44% ± 0.53% R = 87.65% ± 0.24% R = 49.42 ± 0.61% R = 58.0% IC_50_ = 179.5 ± 12 μM, R = 77.20 ± 1.10% IC_50_ = 497.6 ± 24 μM IC_50_ = 683.1 ± 17 μM	Mixed - -	Cell-based assays/ Molecular docking - -	--	[38]
Skin of Alaska pollock (*Theragra chalcograma*)	Collagen	Gel fraction RP-HPLC fraction GSAGPAGPSGPRGP LGDARNSPAPP	R = 85.42 ± 0.11% R = 92.24 ± 0.36% IC_50_ = 0.166 mM, R = 91.41% IC_50_ = 0.177 mM, R = 88.52%	Mixed +	Molecular docking +	- - SHRs experiment +	[24]
Skin of Alaska pollock (*Gadus chalcogrammus*)	Collagen	Ultrafiltration fraction Gel filtration fraction GPLGVP	IC_50_ = 1.59 ± 0.008 mg/mL 0.27 mg/mL IC_50_ = 105.8 μM	Non-competitive	Molecular docking	--	[39]
Skin of Alaska pollock (*Gadus chalcogrammus*)	Collagen	Hydrolysate	R = 79.62%	--	--	--	[21]
Muscle of lizardfish (*Saurida elongata*)	Myosin & actin	VYP	IC_50_ = 0.045 mg/mL (108 μM)	--	Molecular docking	--	[57]
Scale gelatin from lizardfish (*Saurida elongata*)	Collagen	Hydrolysate Gel fraction AGPPGSDGQPGAK	R = 86.0 ± 0.4% R = 87.6 ± 0.6% IC_50_ = 420 ± 20 μM	--	--	SHRs experiment - -	[54]
Muscle of European pilchard (*Sardina pilchardus*)	Myosin & actin	Hydrolysate Ultrafiltration fraction FIGR FILR FQRL FRAL KLF KFL	IC_50_ = 96.09 ± 9.59 µg/mL IC_50_ = 7.88 ± 1.50 µg/mL IC_50_ = 0.60 ± 0.01 mg/mL IC_50_ = 1.01 ± 0.02 mg/mL IC_50_ = 1.54 ± 0.14 mg/mL IC_50_ = 0.66 ± 0.09 mg/mL IC_50_ = 0.89 ± 0.01 mg/mL IC_50_ = 0.61 ± 0.02 mg/mL	Competitive + - - - -	Molecular docking + - - - -	--	[40]
Muscle of hairtail (*Trichiurus lepturus*)	Myosin & actin	Hydrolysate Ultrafiltration fraction Gel fraction SLAGLP LGGGPI	R = 77.48 ± 1.03% IC_50_ = 2.44 ± 0.13 mg/mL, R = 78.76 ± 1.23% IC_50_ = 1.41 ± 0.06 mg/mL, R = 55.91 ± 3.99% - -	--	Molecular docking +	--	[52]
Muscle of hairtail (*Trichiurus lepturus*)	Myosin	Hydrolysate Ultrafiltration fraction ANSEVAQWR EALVSQLTR	IC_50_ = 268.43 µg/mL IC_50_ = 143.27 µg/mL IC_50_ = 89.58 μM IC_50_ = 91.48 μM	-	Molecular docking +	-	[49]
Muscle of ribbonfish (*Trichiurus lepturus*)	Myosin & actin	Hydrolysate Sephadex G-25 FAGDDAPR QGPIGPR GPTGPAGPR	86.7% 70.0% IC_50_ = 262.98 μM IC_50_ = 81.09 μM IC_50_ = 168.11 μM	Competitive + +	Molecular docking/ Cell-based assays + +	--	[61]
Muscle of Pacific saury (*Cololabis saira*)	Myosin & actin	LEPWR	IC_50_ = 99.5 μM	Mixed	Cell-based assays	SHRs experiment	[48]
Muscle of chub mackerel (*Scomber japonicus*)	Myosin & actin	PLITT	IC_50_ = 48.73 ± 7.59 μM	Mixed	QSAR/ Molecular docking	-	[50]
By-products (muscle) of Spanish mackerel (*Scomberomorus niphonius*)	Myosin & actin	Ultrafiltration fraction DEAE fraction Gel fraction HPLC fraction	R = 45.6% R = 50.3% R = 83.4% R = 87.6%	--	--	--	[62]
Muscle of longsnout catfish (*Leiocassis longirostris*)	Myosin & actin	Hydrolysate Ultrafiltration fraction Resin fraction Gel fraction	R = 18.7% R = 35.4% R = 56.6% R = 66.9%	--	--	--	[22]
Swim bladder of bighead croaker (*Miichthys miiuy*)	Collagen	Hydrolysate MSCH-1 MSCH-1b SPYGF SHGEY VIGPF GPFGTD GPWGPA DEGPE EVGIQ	R = 51.37 ± 2.06% R = 62.9 ± 2.39% R = 76.25 ± 3.61% IC_50_ = 0.37 ± 0.06 mg/mL IC_50_ = 0.86 ± 0.12 mg/mL IC_50_ = 1.32 ± 0.17 mg/mL IC_50_ = 1.65 ± 0.13 mg/mL IC_50_ = 2.18 ± 0.24 mg/mL IC_50_ = 2.79 ± 0.42 mg/mL IC_50_ = 4.06 ± 0.37 mg/mL	--	Cell-based assays/ Molecular docking - - - - - -	--	[53]
Skin of tawny puffer (*Takifugu flavidus*)	Collagen	Hydrolysate Ultrafiltration fraction HPLC fraction Gel fraction PPLLFAAL	IC_50_ = 0.1 mg/mL IC_50_ = 0.58 mg/mL R = 90% IC_50_ = 0.34 mg/mL IC_50_ = 0.24 mg/mL (28 μM)	Non-competitive	Molecular docking	SHRs experiment	[46]
Swim bladder of monkfish (*Lophius litulon*)	Collagen	Hydrolysate Ultrafiltration fraction Gel fraction RP-HPLC fraction: -MHP6 -MHP7 -MHP9 SEGPK FDGPY SPGPW	R = 53.22 ± 2.63% R = 62.75 ± 1.89% R = 76.81 ± 2.76% R = 86.34 ± 3.25% R = 84.99 ± 2.34% R = 5.39 ± 3.46% IC_50_ = 0.63 mg/mL (1219.7 μM) IC_50_ = 0.94 mg/mL (1573.0 μM) IC_50_ = 0.71 mg/mL (1308.5 μM)	--	Molecular docking/ Cell-based assays	--	[8]
Muscle of large yellow croaker (*Larimichthys crocea*)	Myosin	Ultrafiltration fraction IPYADFK LYDHLGK INEMLDTK IHFGTTGK NWPWMK FYEPFM	IC_50_ = 0.26 mg/mL, R = 96.29% IC_50_ = 0.63 ± 0.09 μM IC_50_ = 9.41 ± 0.24 μM IC_50_ = 6.53 ± 0.21 μM IC_50_ = 5.34 ± 0.15 μM IC_50_ = 6.23 ± 0.34 μM IC_50_ = 10.26 ± 0.21 μM	Competitive + + + + Non-competitive	QSAR/ Molecular docking + + + + +	--	[43]
Muscle of large yellow croaker (*Larimichthys crocea*)	Titin	WAR WQR	IC_50_ = 31.2 ± 0.8 μM IC_50_ = 231.33 ± 0.02 μM	non-competitive -	Molecular docking -	--	[44]
By-products (bone, skin and tail) of shortfin scad (*Decapterus macrosoma*)	Mixture	Hydrolysate Ultrafiltration fraction Gel fraction RGVGPVPAA	IC_50_ = 3.98 mg/mL, R = 79.34 ± 0.79% IC_50_ = 2.20 mg/mL, R = 81.50 ± 0.26% IC_50_ = 1.59 mg/mL, R = 81.47 ± 0.41% IC_50_ = 0.20 mg/mL (244.5 μM), R = 87.99 ± 2.79%	Competitive	Molecular docking	--	[63]
Skin of cobia (*Rachycentron canadum*)	Collagen	Hydrolysate in vitro digestion fraction Gel fraction WAA AWW IWW WL	IC_50_ = 0.221 mg/mL IC_50_ = 0.304 mg/mL IC_50_ = 0.020 mg/mL IC_50_ = 118.50 μM IC_50_ = 9.40 μM IC_50_ = 0.51μM IC_50_ = 26.80 μM	--	--	SHRs experiment - - - - - -	[47]

Note: / means and, + means the same as the above, - means non, -- means all non.

## Data Availability

Data sharing does not apply to this article, as no datasets were generated or analyzed during the current study.
